# Remote Sulfonylation of Anilines with Sodium Sulfifinates Using Biomass-Derived Copper Catalyst

**DOI:** 10.3390/molecules29204815

**Published:** 2024-10-11

**Authors:** Xiaoping Yan, Jinguo Wang, Chao Chen, Kai Zheng, Pengfei Zhang, Chao Shen

**Affiliations:** 1 Key Laboratory of Pollution Exposure and Health Intervention of Zhejiang Province, College of Biology and Environmental Engineering, Zhejiang Shuren University, Hangzhou 310015, China; 2 Key Laboratory of Organosilicon Chemistry and Material Technology, College of Material, Chemistry and Chemical Engineering, Ministry of Education, Hangzhou Normal University, Hangzhou 311121, China

**Keywords:** biomass derivation, sulfonylation, heterogeneous, C-H activation

## Abstract

A biomass-based catalyst, Cu_x_O_y_@CS-400, was employed as an excellent recyclable heterogeneous catalyst to realize the sulfonylation reaction of aniline derivatives with sodium sulfinates. Various substrates were compatible, giving the desired products moderate to good yields at room temperature. In addition, this heterogeneous copper catalyst was also easy to recover and was recyclable up to five times without considerably deteriorating in catalytic efficiency. Importantly, these sulfonylation products were readily converted to the corresponding 4-sulfonyl anilines via a hydrolysis step. The method offers a unique strategy for synthesizing arylsulfones and has the potential to create new possibilities for developing heterogeneous copper-catalyzed C-H functionalizations.

## 1. Introduction

**Figure 1:**
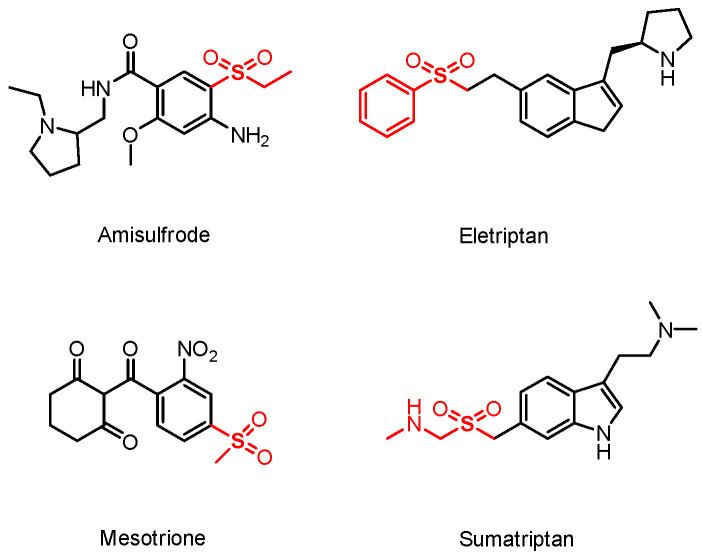
Examples of aryl sulfone-based bioactive molecules.

Sulfone is widely utilized in diverse fields such as agricultural chemistry, medicine, or advanced materials due to its notable chemical, pharmaceutical, and biological activities [1,2,3]. Some biologically active aryl sulfones are shown in Figure 1. Therefore, a significant amount of research has been focused on the synthesis of aryl sulfones through a variety of pathways, including sulfide oxidation, sulfinate alkylation, arene sulfonylation of the Friedel–Crafts type, and electrophilic substitution of aromatics with sulfonyl halides or sulfonic acids [4,5,6,7].

**Scheme 1:**
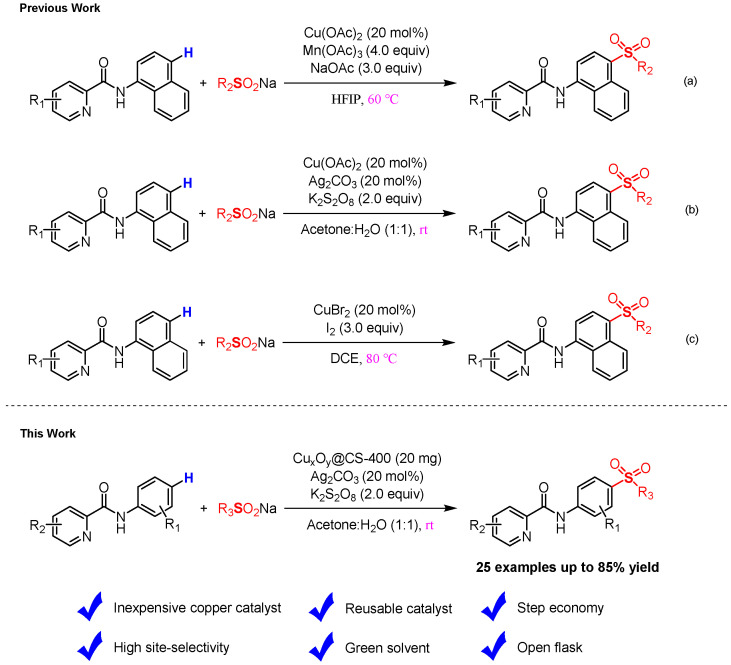
Direct sulfonylation of 1-naphthylamides or anilines at the C4 site.

In recent years, the formation of transition metal-catalyzed C-H functional syntheses of sulfur-containing compounds by direct C-S bonding has proven to be one of the most effective protocols, because this strategy makes the C-S cross-coupling simpler and more efficient [8]. Traditional aromatic sulfonation reactions usually require metal catalysts and chemical oxidants [9,10,11,12,13]. For example, Manolikakes’s group and Wu’s group have independently mentioned the remote para-selective sulfonylation of 1-naphthylamides using Cu(OAc)_2_-catalyzed sodium sulfinates (Scheme 1a,b) [14,15]. Xiong’s group developed the use of CuBr_2_ as a catalyst for highly selective functionalization of the C4-H position of 1-naphthylamides using sodium sulfinates (Scheme 1c) [16]. However, most copper catalysts that are used are homogeneous and not environmentally friendly. Consequently, creating a green reaction pathway to satisfy this requirement for sulfonylation remains significant and difficult.

Compared to homogeneous catalysts [17,18,19,20,21,22,23,24,25], heterogeneous catalysts provide the advantage of being able to be retrieved from reaction mixtures and reused, resulting in reduced economic and environmental expenses [26,27,28,29,30]. Biomass-derived heterogeneous catalysts are garnering an increasing amount of attention [31,32,33,34]. Various organic reactions for heterogeneous biomass-based catalysts have been reported by our group in recent years [35,36,37,38,39]. The biomass resources used for these catalyst materials have the advantages of leading to high-efficiency catalysts, being low-cost materials, and requiring a simple preparation process. Herein, we report the synthesis of a biomass-based copper catalyst and its successful application in the sulfonylation of aniline derivatives with sodium sulfinates, producing sulfonylation products in acceptable to good yields (Scheme 1c). 

## 2. Results and Discussion

**Scheme 2:**
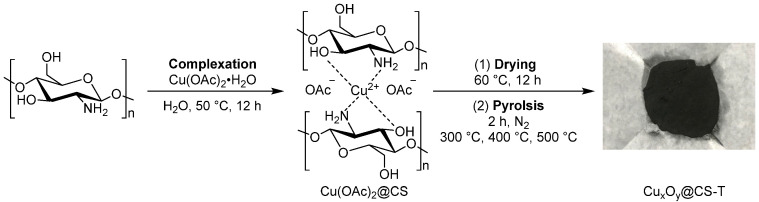
Synthesis of Cu_x_O_y_@CS-T catalyst.

According to our previous synthesis method [36], the Cu_x_O_y_@CS-T catalyst was synthesized using a sequential approach outlined in Scheme 2, where T represents the pyrolysis temperature. Firstly, a straightforward and practical method was used to create the catalyst precursor, which was a metal–chitosan complex obtained by combining Cu(OAc)_2_ with commercial chitosan in water at 50 °C. Then, the catalyst precursor underwent drying at 60 °C and pyrolysis at high temperatures (300 °C, 400 °C, and 500 °C), which formed the Cu_x_O_y_@CS-T catalyst. Since the catalyst is stable to air and moisture both in the solid form and in a solution, it was kept at room temperature in a screw-capped vial without any additional air protection. 

**Figure 2:**
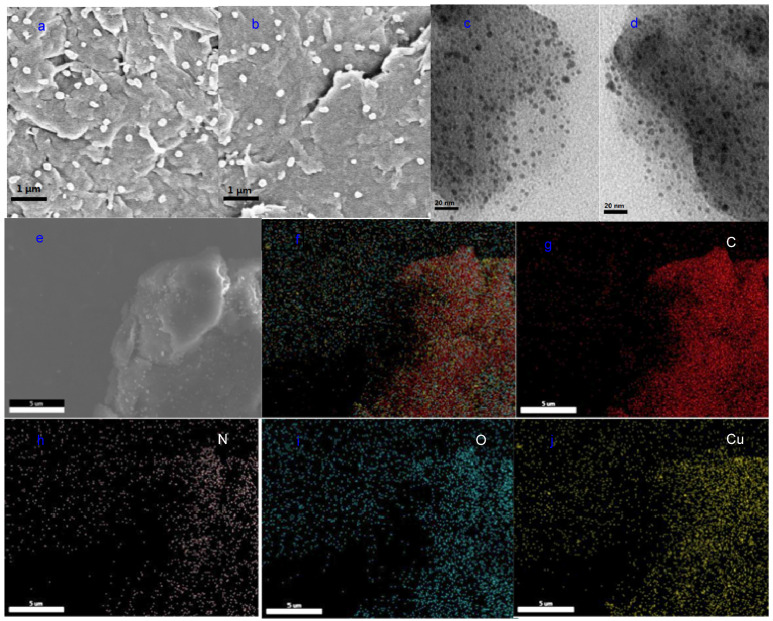
SEM images of Cu_x_O_y_@CS-400 (**a**,**b**). TEM images of Cu_x_O_y_@CS-400 (**c**,**d**). Elemental mapping of Cu_x_O_y_@CS-400 (**e**–**j**).

A scanning electron microscope (SEM) (Figure 2a,b) and a transmission electron microscope (TEM) (Figure 2c,d)were used to examine the Cu_x_O_y_@CS-400 catalyst. As shown in Figure 2, the copper particles were evenly distributed on the catalyst carrier surface, and a size of approximately 0.3–0.5 um was obtained. The structural characterization showed that the copper was evenly distributed on the biochar without obvious agglomeration. To confirm the element composition of the Cu_x_O_y_@CS-400, energy-dispersive spectroscope (EDS) element mapping was conducted (Figure 2e,j). The element mapping demonstrated the uniform dispersion of carbon, nitrogen, and oxygen in conjunction with copper components. In addition, the catalyst was characterized using X-ray diffraction (XRD) and photoelectron spectroscopy (XPS). Our previous studies have shown that the catalyst features three components, Cu, Cu_2_O, and CuO (Figure S1) [36].

With the characterized catalysts in hand [31], we started the investigation by examining the response of *N*-(*o*-tolyl)picolinamide (**1a**) with sodium benzenesulfinates (**2a**) as a model reaction to adjust the reaction conditions (Table 1). We were pleased to discover that by using Cu_x_O_y_@CS-400 as a catalyst at room temperature in a mixed solvent (acetone/H_2_O = 1:1), the expected sulfonylation yield of 82% could be achieved (Table 1, entry 1). Encouraged by this result, the reaction conditions for sulfonylation were modified by adjusting the metal catalysts, silver cocatalysts, oxidants, solvents, and temperatures. An investigation was conducted to analyze the impact of several copper catalysts on the model reaction (Table 1, entries 1–5). It is important to highlight that the reaction did not occur in the absence of a copper catalyst, indicating the unique catalytic function of copper in this process (Table 1, entry 6). The combination of Ag-peroxydisulfate has shown significant efficacy in several sulfonylated reactions [3,15,36]. Therefore, screening of several silver salts showed that the yield of **3a** could not be improved by using either AgNO_3_ or AgOAc instead of Ag_2_CO_3_, whereas no reaction occurred in the presence of AgSbF_6_ (Table 1, entries 7–9). Subsequently, other oxidants such as Na_2_S_2_O_8_, (NH_4_)_2_S_2_O_8_, TBHP, and H_2_O_2_ were investigated (Table 1, entries 10–13), and it was found that K_2_S_2_O_8_ was the most efficient. When Ag_2_CO_3_ or K_2_S_2_O_8_ was not present, the reaction did not produce any product (Table 1, entries 14 and 15). These findings suggest that organic transformation is determined by the Ag_2_CO_3_ and K_2_S_2_O_8_ synergistic action. Then, other solvents (acetone, H_2_O, EtOH, DMSO) were also evaluated (Table 1, entries 16–19), and a mixed solvent of acetone and H_2_O turned out to enhance the yield. In order to further increase the yield of the reaction, we explored the impact of temperature on the reaction outcomes. Changing the temperature did not improve the yield of the sulfonylation product (Table 1, entry 20).

**Scheme 3:**
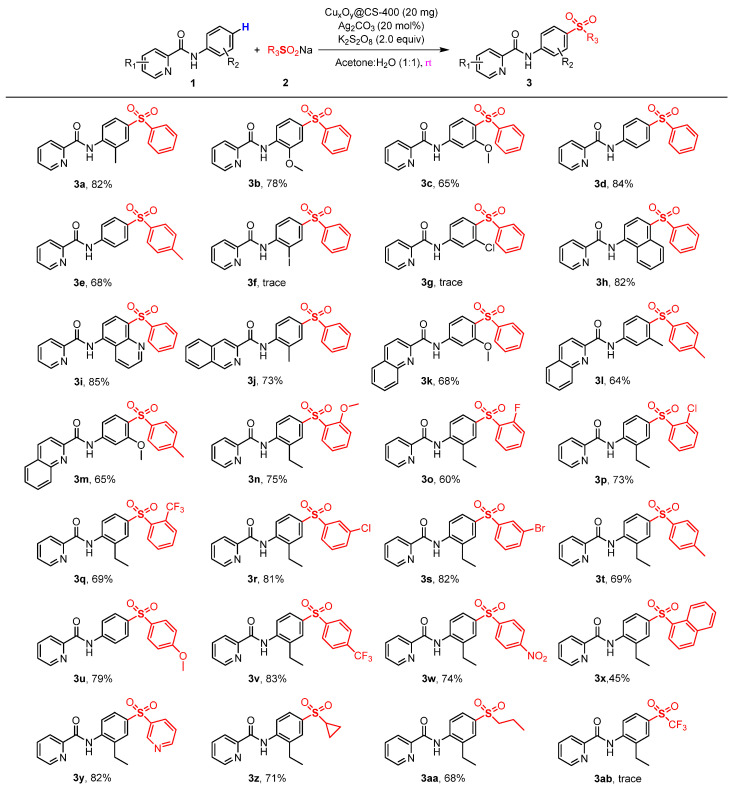
Substrate scope of anilines and sodium sulfonates. Reaction conditions: anilines (0.2 mmol), sodium sulfonates (2.0 equiv.), Cu_x_O_y_@CS-400 (20 mg.), Ag_2_CO_3_ (20 mol%), K_2_S_2_O_8_ (2.0 equiv.), acetone/H_2_O (1:1) (3 mL), stirred at rt, under air, 12 h. Isolated yields.

After obtaining the most suitable reaction conditions, we proceeded to investigate the various anilines that could be utilized for sulfonylation (Scheme 3). Substrates bearing an electron-donating substituent at the C2 and C3 positions of the aniline ring were well tolerated (**3a**–**3c**). Changing substituents to unsubstituted aniline could also smoothly facilitate the reaction to obtain **3d** and **3e** in 84% and 68% yields, respectively. Notably, when the substrates containing electron-withdrawing groups (-Cl, -I) were used, the reaction failed to occur (**3f** and **3g**). In addition, 1-naphthylamide and quinolin-5-amine could also provide the required product in a high yield (**3h** and **3i**). The further application of this method focused on the substituted picolinamide moiety. When the picolinamide moiety was varied, products **3j**–**3m** were smoothly obtained. Subsequently, we conducted further research on the wide range of sodium sulfinates. Our catalytic system was highly compatible with functionalized sodium sulfinates, resulting in sulfonylation products with yields ranging from moderate to good. In particular, comparable compounds (**3n**–**3w**) were produced in 60–83% yields by benzene sodium sulfinates with either electron-donating or electron-withdrawing groups at the C2, C3, or C4 sites. Reactions of *N*-(2-ethylphenyl)picolinamide with both naphthalene, heterocyclic, and aliphatic sodium sulfinates proceeded successfully as well, resulting in the formation of corresponding products (**3x**–**3aa**) in 45–82% yields. Unfortunately, sodium trifluoromethanesulfinate could not be converted into **3ab** smoothly. 

**Scheme 4:**
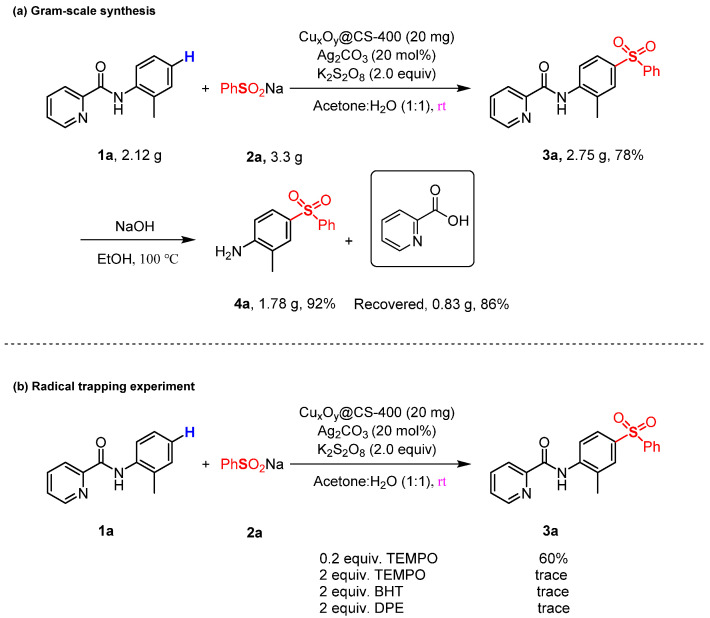
Synthetic applications and control experiment. (**a**) Gram-scale synthesis; (**b**) Radical trapping experiment.

With the goal of confirming the effectiveness and applicability of this approach, we conducted a large-scale reaction (Scheme 4a), resulting in the production of C4 sulfonylation product **3a** with a yield of 78% using the standard conditions. After that, the directing group could be removed by hydrolysis to give 2-methyl-4-(phenylsulfonyl)aniline and picolinic acid in 92% and 82% yields, respectively. In addition, we conducted a few radical trapping studies to further explain the reaction mechanism (Scheme 4b). The addition of the radical inhibitors TEMPO ((2,2,6,6-tetramethylpiperidin-1-yl)oxyl), BHT (butylated hydroxytoluene), or DPE (1,1-diphenylethene) somewhat hindered the sulfonylation process. The reaction was hindered by radical scavengers, indicating the participation of a free radical pathway in the reaction mechanism.

**Scheme 5:**
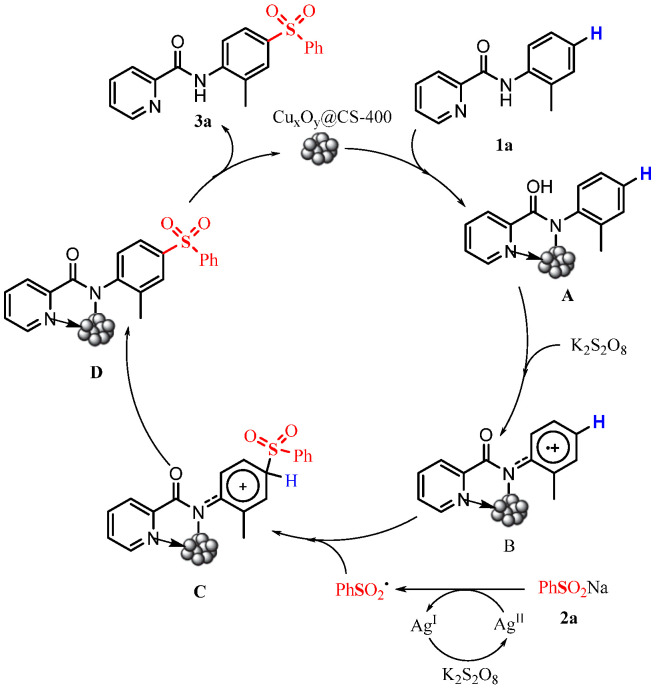
Proposed mechanism.

On the basis of the abovementioned investigations, we suggest that the sulfonylation of anilines with sodium sulfinate occurs by the mechanism described in Scheme 5 [14,15,16,40]. Firstly, the Cu_x_O_y_@CS-400 catalyst and *N*-(o-toly)picolinamide (**1a**) create an aninicimidaate–copper complex **A**. The complex **A** took place as an intermolecular single electron transfer (SET) between the o-toluidine and K_2_S_2_O_8_, furnishing the radical complex **B**. Meanwhile, the benzene sulfonyl radical was produced by reacting sodium benzenesulfinate (**2a**) with silver salts and oxidants. Next, the formation of complex **C** occurred by a radical coupling reaction between radical complex B and the benzenesulfonyl radical. Ultimately, complex **C** undergoes a proton transfer process to form complex **D**, whose dissociation yields the intended product **3a** and involves catalyst regeneration to complete the catalytic cycle.

**Figure 3:**
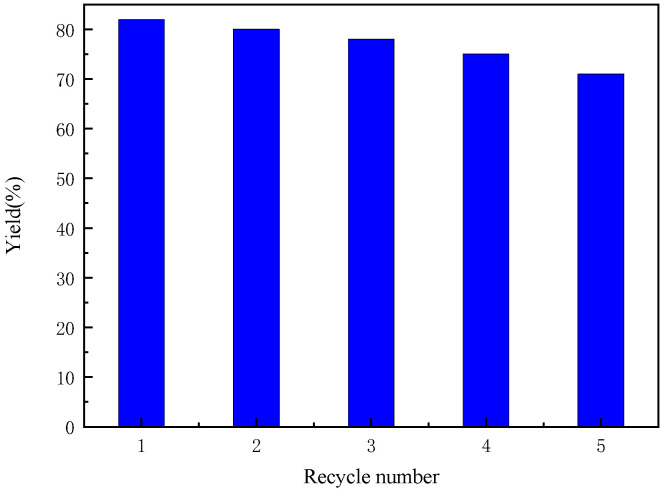
Catalyst reutilization studies.

The appealing elements of heterogeneous catalysis include the use of milder reaction conditions and the option of reusing catalysts. In order to assess the effectiveness and demonstrate the benefits of this approach, the viability of reusing the catalyst was further investigated. After the reaction, the Cu_x_O_y_@CS-400 catalyst was simply recovered by filtration and washed with H_2_O and EtOH. It was reused in the sulfonylation reaction of *N*-(*o*-tolyl)picolinamide and benzenesulfinate, and the results are shown in Figure 3. For the recycling experiment, the Cu_x_O_y_@CS-400 catalyst that had been separated was recharged with a new substrate to be used again in the following run while keeping the same reaction conditions. It was observed that the catalyst maintained its catalytic activity even after being reused five times.

## 3. Experimental Section 

### 3.1. Chemicals and Materials

Chitosan powder (MW: 10,000–50,000, deacetylation degree 95%, purchased from Aladdin reagent (Shanghai) Co., Ltd., Shanghai, China) was used without further purification. Acetophenone and sulfinic acid salts were purchased from Alfa Aesar. Other chemicals were obtained commercially and used without any prior purification. ^1^H NMR spectra were recorded on a Bruker AvanceII 400 spectrometer using TMS as the internal standard. All products were isolated by short chromatography on a silica gel (200–300 mesh) column using petroleum ether (60–90 °C) unless otherwise noted. All compounds were characterized by ^1^H NMR, ^13^C NMR.

### 3.2. General Procedure for Synthesis of Biomass-Derived Copper Catalysts

We dissolved anhydrous copper acetate (90.83 mg, 0.500 mmol) in H_2_O (40 mL) in a 100 mL round-bottom flask equipped with a reflux condenser and magnetic stir bar. Then, chitosan (690 mg) was added to obtain a suspension, which was stirred at 50 °C for 3 h. After the solution was cooled to room temperature, H_2_O was slowly removed under reduced pressure. The light-blue solid obtained was dried under vacuum at 60 °C for 12 h. We transferred the dried sample to a porcelain boat and placed it in the oven. The oven was evacuated and purged with nitrogen for 30 min. Then, we heated the oven to the appropriate temperature (e.g., 300 °C, 400 °C, 500 °C) with a temperature gradient of 2 °C/min and maintained the same temperature under a nitrogen atmosphere for 2 h. Then, we let the oven cool to room temperature. Throughout the process, the furnace was continuously purged with nitrogen. The prepared catalysts were stored in screw-cap vials at room temperature without special air protection.

### 3.3. General Procedure for C–H Sulfonylation of Anilines Derivatives 

We used a 25 mL Schlenk tube equipped with a magnetic stir bar and added *N*-phenylpicolinamide derivative **1** (0.2 mmol), sodium sulfinate **2** (0.4 mmol), Cu_x_O_y_@CS-400 (20 mg), Ag_2_CO_3_ (20 mol%), and K_2_S_2_O_8_ (2.0 equiv.) in acetone/H_2_O (1:1) (3.0 mL). The resulting mixture was stirred at room temperature for 3 h in air. After completion, the mixture was added to H_2_O (20 mL) and extracted three times with ethyl acetate (10 mL). The combined organic layer was dried over anhydrous Na_2_SO_4_ and filtered. After the solvent was evaporated in a vacuum, the residue was purified by silica gel column chromatography using petroleum ether/ethyl acetate as a detergent to obtain pure product **3**.

## 4. Conclusions 

In conclusion, the remote C–H sulfonylation of anilines with sodium sulfinate at room temperature was facilitated by a biomass-based Cu_x_O_y_@CS-400 catalyst, which could be recycled and produced the required products in moderate to good yields. The technique exhibited many advantages, including straightforward and gentle reaction conditions, a small impact on the environment, reduced energy consumption, and excellent tolerance towards functional groups. In addition, the control experiments demonstrated the involvement of a radical route in the reaction. More importantly, the catalyst was reutilized five times without a considerable deterioration in catalytic efficiency.

## Figures and Tables

**Table 1:**
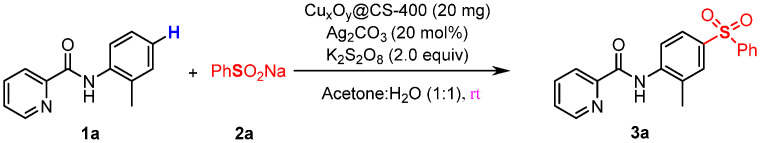
Optimization of reaction conditions ^a^.

Entry	Variation from Standard Conditions	Yield (%) ^b^
1	none	82
2	Cu(OAc)_2_@CS as catalyst	35
3	CuO as catalyst	28
4	Cu_x_O_y_@CS-300 as catalyst	69
5	Cu_x_O_y_@CS-500 as catalyst	65
6	Without CuxOy@CS-400	0
7	AgNO_3_ as cocatalyst	69
8	AgOAc as cocatalyst	73
9	AgSbF_6_ as cocatalyst	0
10	Na_2_S_2_O_8_ as oxidant	76
11	(NH_4_)_2_S_2_O_8_ as oxidant	74
12	TBHP as oxidant	22
13	H_2_O_2_ as oxidant	11
14	Without Ag_2_CO_3_	0
15	Without K_2_S_2_O_8_	0
16	Acetone as solvent	28
17	H_2_O as solvent	54
18	EtOH as solvent	23
19	DMSO as solvent	25
20	60 °C as reaction temperature	72

^a^ Reaction conditions: **1a** (0.2 mmol), **2a** (2.0 equiv.), Cu_x_O_y_@CS-400 (20 mg), Ag_2_CO_3_ (20 mol%), K_2_S_2_O_8_ (2.0 equiv.), acetone/H_2_O =1:1 (3 mL), stirred at rt, under air, 12 h. ^b^ Isolated yields.

## Data Availability

The original contributions presented in this study are included in the article/Supplementary Materials. Further inquiries can be directed to the corresponding author/s.

## Supplementary Materials

The following supporting information can be downloaded at: https://www.mdpi.com/article/10.3390/molecules29204815/s1. Figure S1: XPS spectra of Cu_x_O_y_@CS-400: (a) Survey spectrum. (b) Cu 2p. (c) O 1s. (d) Cu LMM. (e) XRD spectra of Cu_x_O_y_@CS-400. Reference [41] is cited in the supplementary materials.
