# Supplementary material for: Remote Sulfonylation of Anilines with Sodium Sulfifinates Using Biomass-Derived Copper Catalyst
Source: Molecules. 2024 Oct 11;29(20):4815. doi: 10.3390/molecules29204815 (PMC11509939; doi:10.3390/molecules29204815)
Supplement: Supplementary file 1 [file molecules-29-04815-s001.zip › molecules-3184989-supplementary.pdf]

Remote Sulfonylation of Anilines with Sodium Sulfifinates Using Biomass-Derived Copper Catalyst

Xiaoping Yan [1], Jinguo Wang [1], Chao Chen [1,*], Kai Zheng [1], Pengfei Zhang [2] and Chao Shen [1,*]

1 Key Laboratory of Pollution Exposure and Health Intervention of Zhejiang Province, College of Biology and Environmental Engineering, Zhejiang Shuren University, Hangzhou 310015, China

2 Key Laboratory of Organosilicon Chemistry and Material Technology, College of Material, Chemistry and Chemical Engineering, Ministry of Education, Hangzhou Normal University, Hangzhou 311121, China

* Correspondence: chencc@zjsru.edu.cn (C.C.); shenchaozju@zjsru.edu.cn (C.S.)

1. General Information……………………………………………………S2

2. Experimental Section…………………………………………………S2

3. Catalyst Characterization……………………………………………...S3

4. Characterization of the Products.....………………………………........S3

5. Copies of MS spectra..…………………………………………….......S11

6. Copies of NMR spectra….……………………………………….......S13

7. References……………………………………………………………S38

1. General Information

a. Materials

Chitosan powder (MW: 10,000-50,000 deacetylation degree 95%, purchased from Aladdin reagent (Shanghai) Co., Ltd.) was used without further purification. Acetophenone and sulfinic acid salts were purchased from Alfa Aesar. Other chemicals were obtained commercially and used without any prior purification. ^{1}H NMR spectra were recorded on a Bruker AvanceII 400 spectrometer using TMS as the internal standard. All products were isolated by short chromatography on a silica gel (200–300 mesh) column using petroleum ether (60-90 °C) unless otherwise noted. All compounds were characterized by ^{1}H NMR, ^{13}C NMR.

b. Methods

Melting points were determined on an X-5 Data microscopic melting point apparatus. ^{1}H NMR and ^{13}C NMR spectra were recorded on a Bruker Advance 500 spectrometer at ambient temperature with $CDCl_3$ as solvent unless otherwise noted and tetramethylsilane (TMS) as the internal standard. Mass spectra (GC-MS) were acquired on an Agilent 5975 spectrometer. Transmission electron microscopy (TEM) images were taken on the Hitachi HT-7700 microscope. The X-ray diffraction (XRD) data were taken on a German Bruker D8 X-ray diffractometer with Niltered Cu Ka radiation. Thermogravimetric analyses were performed with a Netzsch STA409PC analyzer at 10 °C/min in the air (10 ml/min). 5 mg of each sample in an alumina pan was analyzed in the 30-650 °C temperature range. Analytical thin layer chromatography (TLC) was performed on Merk precoated TLC (silica gel 60 F254) plates.

2. Experimental Section

General procedure for synthesis of biomass-derived copper catalysts[1]:

Dissolve anhydrous copper acetate (90.83 mg, 0.500 mmol) in H_2O (40 mL) in a 100 mL round-bottom flask equipped with a reflux condenser and magnetic stir bar. Then, chitosan (690 mg) was added to obtain a suspension, which was stirred at 50 °C for 3 hours. After the solution was cooled to room temperature, H_2O was slowly removed under reduced pressure. The light blue solid obtained was dried under vacuum at 60°C for 12 hours. Transfer the dried sample to a porcelain boat and place in the oven. The oven was evacuated and purged with nitrogen for 30 minutes. Then, heat the oven to the appropriate temperature (e.g., 300 °C, 400 °C, 500 °C) with a temperature gradient of 2 °C/min and maintain the same temperature under a nitrogen atmosphere for 2 hours.

Then let the oven cool to room temperature. Throughout the process, the furnace was continuously purged with nitrogen. The prepared catalysts were stored in screw-cap vials at room temperature without special air protection.

General procedure for C−H sulfonylation of anilines derivatives [2-4]**:**

Take a 25 mL Schlenk tube equipped with a magnetic stir bar and add *N*-phenylpicolinamide derivative **1** (0.2 mmol), sodium sulfinate **2** (0.4 mmol), Cu_xO_y@CS-400 (20 mg), Ag_2CO_3 (20 mol%), and $K_2S_2O_8$ (2.0 equiv.) in acetone/H_2O(1:1) (3.0 mL). The resulting mixture was stirred at room temperature for 3 hours in air. After completion, the mixture was added to H_2O (20 ml) and extracted three times with ethyl acetate (10 mL). The combined organic layer was dried over anhydrous Na_2SO_4 and filtered. After the solvent was evaporated in a vacuum, the residue was purified by silica gel column chromatography using petroleum ether/ethyl acetate as a detergent to obtain pure product **3**.

3. Catalyst Characterization

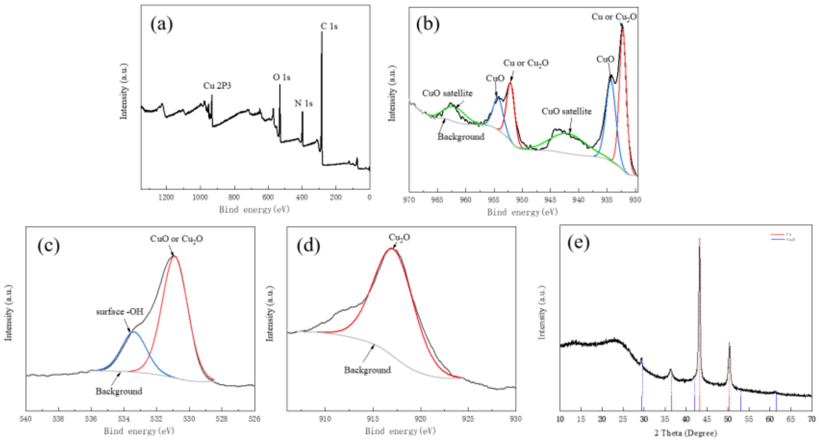

Figure S1. XPS spectra of Cu_xO_y@CS-400:(a) Survey spectrum. (b) Cu 2p. (c) O 1s. (d) Cu LMM. (e) XRD spectra of Cu_xO_y@CS-400.

4. Characterization of the products

***N*-[4-(benzenesulfony)-2-methylphenyl]pyridine-2-carboxamide (3a)**

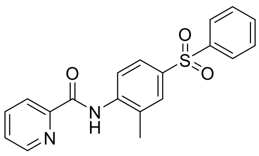

^{1}H NMR (500 MHz, $CDCl_3$) δ 10.41 (s, 1H). δ 8.62 (d, *J* = 4.4 Hz, 1H), 8.54 (d, J = 8.6 Hz, 1H), 8.29 (d, *J* = 7.8 Hz, 1H), 7.94 (t, *J* = 7.2 Hz, 3H), 7.84 (d, *J* = 10.6 Hz, 1H), 7.80 (s, 1H), 7.51 (dt, *J* = 26.6, 7.3 Hz, 5H), 2.46 (s, 3H); ^{13}C NMR

(126 MHz, $CDCl_3$) δ 161.8, 148.9, 147.9, 142.0, 140.5, 136.1, 133.0, 129.6, 129.3, 128.4, 127.5, 127.1, 127.0, 123.0, 120.7, 17.8.

***N*-[4-(benzenesulfony)-2-methoxyphenyl]pyridine-2-carboxamide (3b)** [5]

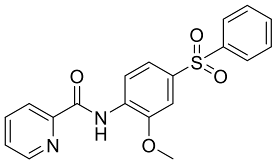

^{1}H NMR (500 MHz, $CDCl_3$) δ 10.74 (s, 1H), 8.77 – 8.75 (d, *J* = 8.5 Hz, 1H), 8.66 (s, 1H), 8.27 (s, 1H), 7.94 (d, *J* = 7.5 Hz, 2H), 7.90 (t, *J* = 7.5 Hz, 1H), 7.61 (dd, *J* = 8.5, 1.4 Hz, 1H), 7.54 (d, *J* = 7.1 Hz, 2H), 7.52 – 7.43 (m, 4H), 4.02 (s, 3H); ^{13}C NMR (126 MHz, $CDCl_3$) δ 148.7, 142.0, 137.7, 135.7, 133.1, 132.1, 129.3, 127.4, 121.7, 119.3, 108.8, 56.4.

***N*-[4-(benzenesulfonyl)-3-methoxyphenyl]pyridine-2-carboxamide (3c)**

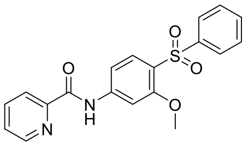

^{1}H NMR (500 MHz, $CDCl_3$) δ 10.48(s, 1H), 8.64 (d, *J* = 4.0 Hz, 1H), 8.33 (d, *J* = 7.6 Hz, 1H), 8.12 (d, *J* = 8.5 Hz, 1H), 8.02 (t, *J* = 7.3 Hz, 1H), 7.96 (d, *J* = 7.4 Hz, 2H), 7.83 (s, 1H), 7.60 (dd, *J* = 6.6, 5.0 Hz. 1H), 7.54 (t, *J* = 7.4 Hz, 1H), 7.46 (t, *J* = 7.7 Hz, 2H), 7.24 (s, 2H), 3.81 (s, 3H); ^{13}C NMR (126 MHz, $CDCl_3$) δ 157.9, 148.1, 147.0, 144.2, 144.2, 141.5, 139.1, 132.8, 130.8, 128. 4, 128.2, 127.3, 124.1, 123.4, 111.0, 103.4, 56.0.

***N*-[4-(benzenesulfonyl)phenyl]pyridine-2-carboxamide (3d)**

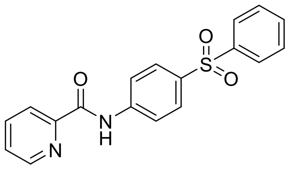

^{1}H NMR (500 MHz, $CDCl_3$) δ 10.52 (s, 1H), 8.65 (d, *J* = 4.4 Hz, 1H), 8.55 (d, *J* = 9.3 Hz, 1H), 8.32 (d, *J* = 7.8 Hz, 1H), 7.96 (dd, *J* = 13.6, 7.6 Hz, 3H), 7.84 (d, *J* = 7.0 Hz, 2H), 7.60-7.43 (m, 5H); ^{13}C NMR (126 MHz, $CDCl_3$) δ 161.7, 149.0, 147.7, 142.1, 139.8, 138.6, 136.5, 134.2, 133.0, 129.3, 127.8, 127.5, 127.1, 127.0, 123.1, 121.2.

***N*-[4-(4-methylbenzenesulfonyl)phenyl]pyridine-2-carboxamide (3e)**

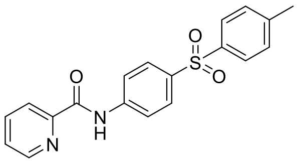

^{1}H NMR (500 MHz, $CDCl_3$) δ 10.52 (s, 1H), 8.64 (d, *J* = 4.6 Hz, 1H), 8.36 (d, *J* = 7.8 Hz, 1H), 8.03 (t, *J* = 7.7 Hz, 1H), 7.96 (s, 1H), 7.95 (s, 1H), 7.63 – 7.59 (m, 1H), 7.29 (d, *J* = 8.2 Hz, 2H). 2.39 (s, 3H); ^{13}C NMR (126 MHz, $CDCl_3$) δ

160.2, 147.2, 145.9, 143.0, 140.9, 138.3, 137.9, 135.9, 132.1, 128.9, 128.3, 128.1, 127.9, 126.5, 126.5, 126.3, 122.6, 118.8, 19.9.

***N*-[4-(benzenesulfonyl)naphthalen-1-yl]pyridine-2-carboxamide (3h)** [6]

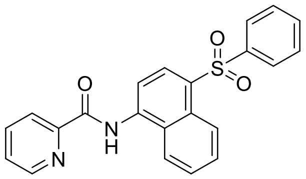

^{1}H NMR (500 MHz, $CDCl_3$) δ 11.15 (s, 1H), 8.71 (d, *J* = 8.2 Hz, 3H), 8.60 (d, *J* = 8.3 Hz, 1H), 8.38 (d, *J* = 7.8 Hz, 1H), 8.17 (d, *J* = 7.8 Hz, 1H), 7.99 (dd, *J* = 17.4, 7.8 Hz, 3H), 7.64 (dd, *J* = 14.3, 7.0 Hz, 2H), 7.61 – 7.57 (m, 1H), 7.52 (t, *J* = 7.3 Hz, 1H), 7.47 (t, *J* = 7.5 Hz, 2H); ^{13}C NMR (126 MHz, $CDCl_3$) δ 162.1, 149.1, 148.0, 142.0, 138.4, 138.3, 132.9, 131.2, 131.1, 129.4, 129.1, 128.3, 127.2, 127.1, 126.1, 125.3, 122.9, 120.9, 115.4.

***N*-[8-(benzenesulfonyl)quinolin-5-yl]pyridine-2-carboxamide (3i)** [6]

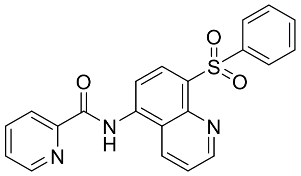

^{1}H NMR (500 MHz, $CDCl_3$) δ 11.07 (s, 1H), 9.0 (d, *J* = 4.1 Hz, 1H), 8.79 (d, *J* = 8.3 Hz, 1H), 8.74 – 8.69 (m, 2H), 8.44 (d, J = 8.6 Hz, 1H), 8.37 (d, *J* = 7.8 Hz, 1H), 8.22 (d, *J* = 8.6 Hz, 2H), 8.00 (t, *J* = 7.7 Hz, 1H), 7.59 (dd, *J* = 7.6, 4.8 Hz, 1H), 7.55 – 7.49 (m, 2H), 7.45 (t, *J* = 7.4 Hz, 2H); ^{13}C NMR (126 MHz, $CDCl_3$) δ 162.1, 150.8, 148.9, 148.1, 144.1, 142.1, 138.3, 138.2, 133.2, 133.0, 132.7, 129.2, 129.0, 127.3, 122.9, 121.6, 121.1, 116.0.

***N*-[4-(benzenesulfonyl)-2-methylphenyl]isoquinoline-3-carboxamide (3j)** [5]

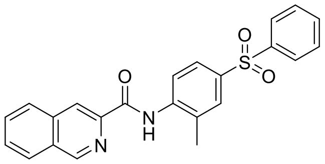

^{1}H NMR (500 MHz, $CDCl_3$) δ 10.73 (s, 1H). δ 8.76 (d, *J* = 8.5 Hz, 1H), 8.65 (d, *J* = 4.1 Hz, 1H), 8.27 (d, *J* = 7.8 Hz, 1H), 7.93 (dd, *J* = 21.8, 7.1 Hz, 4H), 7.61 (d, *J* = 8.5 Hz, 1H), 7.58 – 7.46 (m, 6H), 4.03 (s, 3H); ^{13}C NMR (126 MHz, $CDCl_3$) δ 162.4, 149.5, 148.7, 142.0, 137.8, 135.8, 133.0, 132.0, 129.2, 127.4, 126.8, 122.6. 119.4, 108.8, 56.5.

***N*-[4-(benzenesulfonyl)-3-methoxyphenyl]quinoline-2-carboxamide (3k)** [5]

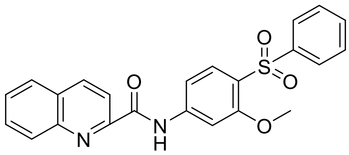

^{1}H NMR (500 MHz, $CDCl_3$) δ 10.51 (s, 1H), 8.41 (d, *J* = 8.5 Hz, 1H), 8.36 (d, *J* = 8.5 Hz, 1H), 8.23 (d, *J* = 8.5 Hz, 1H), 8.17 (d, *J* = 8.6 Hz, 1H),

7.99 (d, *J* = 8.8 Hz, 2H), 7.94 (d, *J* = 5.3 Hz, 2H), 7.66 – 7.82 (m, 1H), 7.69 (t, *J* = 8.0 Hz, 1H), 7.56 (t, *J* = 7.4 Hz, 1H), 7.49 (t, J = 7.7 Hz, 2H), 3.85 (s, 3H); ^{13}C NMR (126 MHz, $CDCl_3$) δ 157.2, 147.6, 143.3, 140.7, 137.4, 131.8, 129.8, 128.5, 127.6, 127.5, 127.2, 126.9, 122.9, 117.6, 110.0, 102.3, 55.0.

***N*-[3-methyl-4-(4-methylbenzenesulfonyl)phenyl]quinoline-2-carboxamide (3l)**

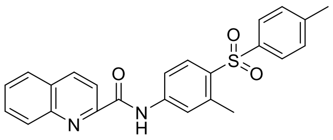

^{1}H NMR (500 MHz, $CDCl_3$) δ 10.51 (s, 1H), 8.40 (d, *J* = 9.1 Hz, 2H), 8.25 (d, *J* = 7.7 Hz, 2H), 7.96 – 7.68 (m, 8H), 7.29 (d, *J* = 6.9 Hz, 1H), 2.48 (s, 3H), 2.41 (s, 3H); ^{13}C NMR (126 MHz, $CDCl_3$) δ 148.6, 145.7, 143.8, 142.2, 139.6, 138.7, 134.3, 130.9, 129.6. 129.3, 128.7, 127.9, 127.6, 122.8. 119.0, 117.0, 21.5, 20.4.

***N*-[3-methoxy-4-(4-methylbenzenesulfonyl)phenyl]quinoline-2-carboxamide (3m)**

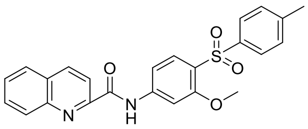

^{1}H NMR (500 MHz, $CDCl_3$) δ 10.52 (s, 1H), 10.86 (s, 1H), 8.51 (d, *J* = 8.5 Hz, 1H), 8.44 – 8.38 (m, 2H), 8.14 (d, *J* = 8.6 Hz, 1H), 7.98 (d, *J* = 8.1 Hz, 1H), 7.93 – 7.83 (m, 4H), 7.74 (t, *J* = 7.5 Hz, 1H), 7.36 (d, *J* = 8.2 Hz, 1H), 7.28 (d, *J* = 8.1 Hz, 2H), 3.85 (s, 3H), 2.41 (s, 3H); ^{13}C NMR (126 MHz, $CDCl_3$) δ 157.1, 147.0, 143.1, 142.6, 138.8, 137.8, 130.7, 129.7, 128.2, 128.1, 127.3, 127.0, 118.3, 110.2, 102.6, 55.0, 20.6.

***N*-[2-ethyl-4-(2-methoxybenzenesulfonyl)phenyl]pyridine-2-carboxamide (3n)**

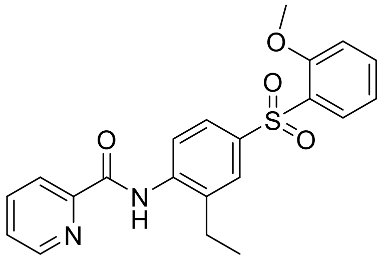

^{1}H NMR (500 MHz, $CDCl_3$) δ 10.47 (s, 1H), 8.67 – 8.55 (m, 2H), 8.29 (d, *J* = 7.4 Hz, 1H), 8.19 (d, *J* = 7.5 Hz, 1H), 7.93 (d, 1H), 7.80 – 7.70 (m, 2H), 7.52 (d, 1H), 7.46 (t, 1H), 7.38 (t, 1H), 7.22 (d, *J* = 7.0 Hz, 1H), 2.88 -2.77 (m, 2H), 2.48 (s, 3H), 1.34 (t, *J* = 7.1 Hz, 3H); ^{13}C NMR (126 MHz, $CDCl_3$) δ 162.1, 149.3, 148.1, 139.78, 139.3, 138.1, 137.8, 135.8, 133.4, 132.7, 129.2, 127.9, 127.7, 127.0, 127.0, 126.5, 122.7, 120.4, 24.4, 20.2, 13.3; ESI–MS: m/z evaluated 396.11, found 397.24 $[M+H]^+$

Table S2. ^{13}C data of compounds **3n**

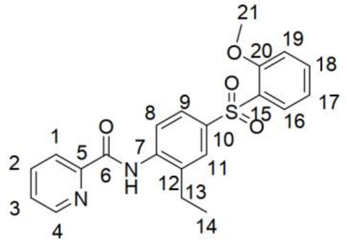

δ_C	**Number**	**Attribution**
162.0	1	6
149.3	1	20
148.1	1	5
139.8	1	4
139.3	1	7
138.1	1	2
137.8	1	10
135.8	1	18
133.4	1	12
132.7	1	16
129.2	1	15
127.9	1	3
127.7	1	11
127.0	1	9
126.9	1	1
126.5	1	17
122.7	1	19
120.4	1	8
24.4	1	21
20.2	1	13
13.3	1	14

***N*-[2-ethyl-4-(2-fluorobenzenesulfonyl)phenyl]pyridine-2-carboxamide (3o)** [5]

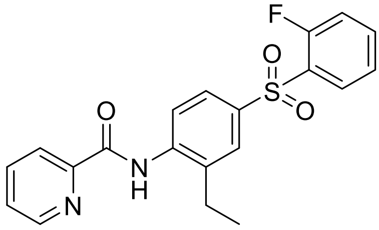

^{1}H NMR (500 MHz, $CDCl_3$) δ 10.48 (s, 1H), 8.63 (d, *J* = 8.5 Hz, 2H), 8.27 (s, 1H), 8.08 (t, *J* = 8.1 Hz, 1H), 7.89 (dd, *J* = 15.8, 9.7 Hz, 3H), 7.55 (dd, *J* = 15.0, 6.9 Hz, 2H), 7.30 (t, *J* = 7.6 Hz, 1H), 7.12 – 7.05 (m, 1H), 2.82 (q, *J* = 7.4 Hz, 2H), 1.36 (t, *J* = 7.5 Hz, 3H); ^{13}C NMR (126 MHz, $CDCl_3$) δ 160.2, 158.1,

140.5, 138.0, 135.8, 135.8, 135.4, 133.5, 129.9, 129.8, 129.6, 128.4, 127.5, 124.6, 124.6, 120.4, 117.4, 117.2; ESI–MS: m/z evaluated 384.42, found 385.22 $[M+H]^+$.

***N*-[4-(2-chlorobenzenesulfonyl)-2-ethylphenyl]pyridine-2-carboxamide (3p)** [5]

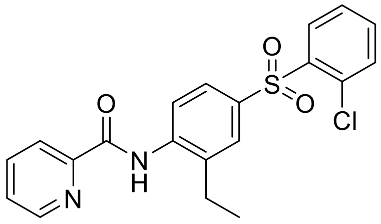

^{1}H NMR (500 MHz, $CDCl_3$) δ 10.50 (s, 1H), 8.63 (d, *J* = 8.5 Hz, 2H), 8.34 (d, *J* = 7.5 Hz, 1H), 8.29 (d, *J* = 7.7 Hz, 1H), 7.93 (t, *J* = 7.6 Hz, 1H), 7.88 (s, 1H), 7.81 (d, *J* = 8.4 Hz, 1H), 7.49 (dd, *J* = 16.0, 8.3 Hz, 3H), 7.42 (d, *J* = 7.2 Hz, 1H), 2.83 (dd, *J* = 14.8, 7.3 Hz, 2H), 1.35 (t, *J* = 7.5 Hz, 3H); ^{13}C NMR (126 MHz, $CDCl_3$) δ 162.1, 149.3, 148.2, 140.3, 138.9, 138.0, 134.5, 134.5, 133.1, 132.8, 132.1, 130.9, 128.9, 127.9, 127.3, 127.0, 122.6, 120.2, 24.4, 13.3; ESI–MS: m/z evaluated 400.06, found 402.86 $[M+2]^+$.

***N*-{2-ethyl-4-[2-(trifluoromethyl)benzenesulfonyl]phenyl}pyridine-2-carboxamide (3q)**

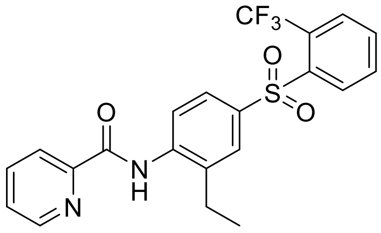

^{1}H NMR (500 MHz, $CDCl_3$) δ 10.45 (s, 1H), 8.60 (d, *J* = 29.7 Hz, 2H), 8.40 (d, *J* = 31.2 Hz, 1H), 8.26 (d, *J* = 22.6 Hz, 1H), 8.03 – 7.59 (m, 6H), 7.49 (d, *J* = 22.4 Hz, 1H), 2.79 (d, *J* = 24.4 Hz, 2H), 1.33 (d, *J* = 23.8 Hz, 3H); ^{13}C NMR (126 MHz, $CDCl_3$) δ 161.9, 149.3, 148.3, 140.5, 140.1, 138.4, 133.4, 133.3, 132.6, 132.3, 128.5, 128.3, 127.4, 127.1, 122.7, 120.4, 24.5, 13.3; ESI–MS: m/z evaluated 434.09, found 436.39 $[M+2]^+$.

***N*-[4-(3-chlorobenzenesulfonyl)-2-ethylphenyl]pyridine-2-carboxamide (3r)** [5]

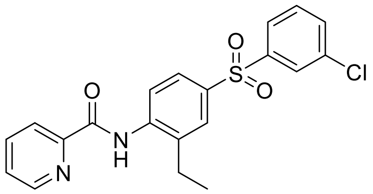

^{1}H NMR (500 MHz, $CDCl_3$) δ 10.49 (s, 1H), 8.64 (t, *J* = 8.6 Hz, 2H), 8.35 (d, *J* = 7.5 Hz, 1H), 8.30 (d, *J* = 7.0 Hz, 1H), 7.93 (dd, *J* = 21.4, 12.0 Hz, 2H), 7.86 – 7.78 (m, 2H), 7.54 – 7.41 (m, 3H), 2.83 (q, *J* = 7.5 Hz, 2H), 2.83 (q, *J* = 7.5 Hz, 2H); ^{13}C NMR (126 MHz, $CDCl_3$) δ 143.9, 138.9, 138.0, 135.4, 134.4, 133.9, 133.1, 132.8, 132.1, 130.9, 130.6, 127.9, 127.5, 127.3, 125.6, 122.7, 120.8, 120.2, 24.5, 13.3.

N-[4-(3-bromobenzenesulfonyl)-2-ethylphenyl]pyridine-2-carboxamide (3s) [5]

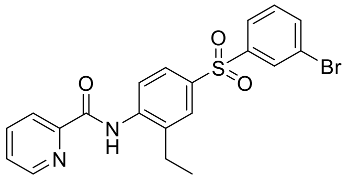

^{1}H NMR (500 MHz, $CDCl_3$) δ 10.49 (s, 1H), 8.65 (d, J = 8.6 Hz, 2H), 8.29 (d, J = 7.6 Hz, 1H), 8.07 (s, 1H), 7.94 (t, J = 7.7 Hz, 1H), 7.88 – 7.79 (m, 3H), 7.66 (d, J = 8.0 Hz, 1H), 7.53 (s, 1H), 7.37 (t, J = 7.9 Hz, 1H), 2.83 (q, J = 7.5 Hz, 2H), 1.37 (t, J = 7.5 Hz, 3H); ^{13}C NMR (126 MHz, $CDCl_3$) δ 162.1, 149.3, 148.3, 144.0, 140.4, 138.0, 136.0, 135.3, 133.9, 130.8, 130.3, 127.9, 127.2, 127.0, 126.1, 123.2, 122.7, 120.7, 24.5, 13.2.

N-[2-ethyl-4-(4-methylbenzenesulfonyl)phenyl]pyridine-2-carboxamide (3t)

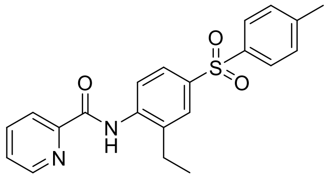

^{1}H NMR (500 MHz, $CDCl_3$) δ 10.45 (s, 1H), 8.64 (d, J = 4.7 Hz, 1H), 8.60 (d, J = 9.0 Hz, 1H), 8.29 (d, J = 7.8 Hz, 1H), 7.93 (t, J = 6.5 Hz, 1H), 7.83 (d, J = 8.5 Hz, 4H), 7.54 – 7.50 (m, 1H), 7.29 (d, J = 8.1 Hz, 2H), 2.81 (q, J = 7.5 Hz,2H), 2.39 (s, 3H), 1.36 (t, J = 7.6 Hz, 3H); ^{13}C NMR (126 MHz, $CDCl_3$) δ 162.0, 149.4, 148.2, 143.9, 139.8, 139.0, 138.0, 136.6, 133.7, 129.9, 127.7, 127.5, 126.9, 122. 7, 120.6.

N-[2-ethyl-4-(4-methoxybenzenesulfonyl)phenyl]pyridine-2-carboxamide (3u)

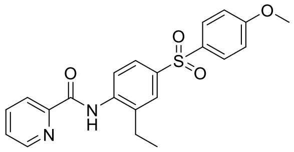

^{1}H NMR (500 MHz, $CDCl_3$) δ 10.45 (s, 1H), 8.63 (d, J = 4.3 Hz, 1H), 8.57 (d, J = 9.3 Hz, 1H), 8.29 (d, J = 7.8 Hz, 1H), 7.94 (t, J = 8.3 Hz, 1H), 7.87 (d, J = 8.9 Hz, 2H), 7.80 (d, J = 7.0 Hz, 2H), 7.54 – 7.50 (m, 1H), 6.95 (d, J = 8.9 Hz, 2H), 3.83 (s, 3H), 2.81 (q, J = 7.5 Hz, 2H), 1.35 (t, J = 7.6 Hz, 3H); ^{13}C NMR (126 MHz, $CDCl_3$) δ 166.1, 163.2, 162.7. 149.3, 148.1, 139.6, 138.1, 137.1, 133.8, 133.6, 129.7, 127.5, 127.0, 126.6, 122.7, 120.8, 115.1, 114.5, 55.1, 24.5, 13.3.

N-{2-ethyl-4-[4-(trifluoromethyl)benzenesulfonyl]phenyl}pyridine-2-carboxamide (3v) [5]

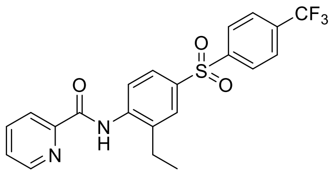

^{1}H NMR (500 MHz, $CDCl_3$) δ 10.50 (s, 1H), 8.65 (d, J = 8.5 Hz, 2H), 8.30 (d, J = 7.4 Hz, 1H), 8.07 (d, J = 8.4 Hz, 2H), 7.96 (t, J = 7.6 Hz, 1H), 7.88 – 7.81 (m, 2H), 7.76 (d, J = 8.4 Hz, 2H), 7.54 (s, 1H), 2.83 (q, J = 7.5 Hz, 2H),

1.37 (t, J = 7.5 Hz, 3H); ^{13}C NMR (126 MHz, $CDCl_3$) δ 162.1, 149.2, 148.2, 145.7, 140.6, 138.2, 135.0, 134.0, 128.0, 127.1, 126.4, 124.3, 122.8, 122.1, 120.8, 24.4, 13.2.

***N*-[2-ethyl-4-(4-nitrobenzenesulfonyl)phenyl]pyridine-2-carboxamide (3w)**

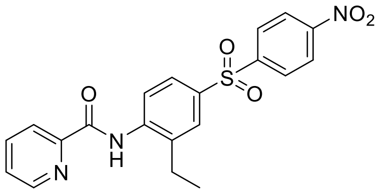

^{1}H NMR (500 MHz, $CDCl_3$) δ 10.52 (s, 1H), 8.75 – 8.58 (m, 2H), 8.31 (dd, J = 20.6, 7.1 Hz, 3H), 8.12 (d, J = 7.5 Hz, 2H), 7.94 (s, 1H), 7.87 (d, J = 7.4 Hz, 2H), 7.53 (s, 1H), 2.84 (d, J = 6.4 Hz, 2H), 1.37 (s, 3H); ^{13}C NMR (126 MHz, $CDCl_3$) δ 162.2, 150.2, 149.2, 148.3, 147.9, 140.9, 138.0, 134.22, 134.0, 128.8, 128.1, 127.5, 127.1, 124.5, 122.7, 120.7, 29.7, 24.4, 13.2.

***N*-[2-ethyl-4-(naphthalene-1-sulfonyl)phenyl]pyridine-2-carboxamide (3x)**

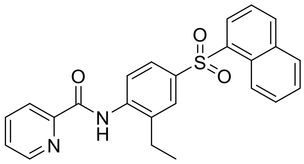

^{1}H NMR (500 MHz, $CDCl_3$) δ 10.49 (s, 1H), 8.64 (d, J = 4.3 Hz, 1H), 8.61 (d, J = 8.6 Hz, 1H), 8.58 (s, 1H), 8.31 (d, J = 7.9 Hz, 1H), 7.99 (d, J = 8.9 Hz, 1H), 7.91 (ddd, J = 24.9, 14.1, 7.4 Hz, 6H), 7.65 – 7.58 (m, 2H), 7.55 – 7.52 (m, 1H), 2.83 (q, J = 7.5 Hz, 2H), 1.36 (t, J = 7.6 Hz, 3H); ^{13}C NMR (126 MHz, $CDCl_3$) δ 161.9, 149.2, 148.0, 139.9, 138.9, 138.2, 136.3, 134.9, 133.8, 132.3, 129.6, 129.4, 129.0, 128.8, 127.9, 127.6, 127.1, 127.0, 122.9, 122.7, 120.8, 30.3, 24.5.

***N*-[2-ethyl-4-(pyridine-3-sulfonyl)phenyl]pyridine-2-carboxamide (3y)**

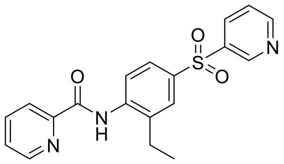

^{1}H NMR (500 MHz, $CDCl_3$) δ 10.51 (s, 1H), 9.16 (s, 1H), 8.78 (s, 1H), 8.69 (d, J = 4.9 Hz, 1H), 8.64 (d, J = 4.7 Hz, 1H), 8.28 (d, J = 7.8 Hz, 2H), 7.94 (t, J = 8.4 Hz, 1H), 7.88 – 7.82 (m, 2H), 7.52 (dd, J = 12.3, 7.3 Hz, 2H), 2.83 (q, J = 7.5 Hz, 2H), 1.37 (t, J = 7.5 Hz, 3H); ^{13}C NMR (126 MHz, $CDCl_3$) δ 162.2, 152.4, 149.2, 148.3, 147.7, 140.8, 139.3, 138.0, 136.0, 134.8, 134.0, 128.0, 127.3, 127.0, 124.3, 122.7, 120.8, 24.5, 13.2.

***N*-[4-(cyclopropanesulfonyl)-2-ethylphenyl]pyridine-2-carboxamide (3z)** [5]

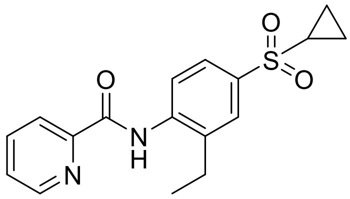

^{1}H NMR (500 MHz, $CDCl_3$) δ 10.51 (s, 1H), 8.67 – 8.62 (m, 2H), 8.33 (d, *J* = 7.8 Hz, 1H), 7.96 (t, *J* = 8.4 Hz, 1H), 7.82 – 7.75 (m, 2H), 7.57 – 7.52 (m, 1H), 2.86 (q, *J* = 7.5 Hz, 2H), 1.39 (t, *J* = 7.6 Hz, 3H), 1.35 (dd, *J* = 4.7, 2.0 Hz, 2H), 1.06 – 0.99 (m, 2H); ^{13}C NMR (126 MHz, $CDCl_3$) δ 162.1, 149.3, 148.1, 140.1, 138.2, 135.6, 133.8, 127.8, 127.0, 126.8, 122.8, 120.8, 33.2, 24.5, 13.3, 6.0; ESI–MS: m/z evaluated 330.10, found 331.26 $[M+H]^+$.

***N*-[2-ethyl-4-(propane-1-sulfonyl)phenyl]pyridine-2-carboxamide (3aa)** [5]

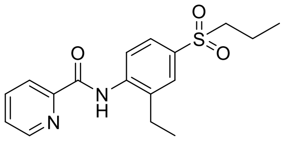

^{1}H NMR (500 MHz, $CDCl_3$) δ 10.51 (s, 1H), 8.67 (d, *J* = 11.1 Hz, 2H), 8.32 (d, *J* = 7.3 Hz, 1H), 7.95 (t, *J* = 7.0 Hz, 1H), 7.85 – 7.73 (m, 2H), 7,54 (s, 1H), 3.14 – 3.01 (m, 2H), 2.86 (dd, *J* = 14.1, 6.8 Hz, 2H), 1.76 (dd, *J* = 14.9, 7.4 Hz, 2H), 1.39 (t, *J* = 7.3 Hz, 3H), 1.00 (t, *J* = 7.2 Hz, 3H); ^{13}C NMR (126 MHz, $CDCl_3$) δ 162.2, 149.4, 148.3, 140.3, 138.0, 133.6, 128.2, 127.3, 127.0, 122.7, 120.6, 58.3, 24.5, 16.6, 13.3, 13.0.

5. Copies of MS spectra

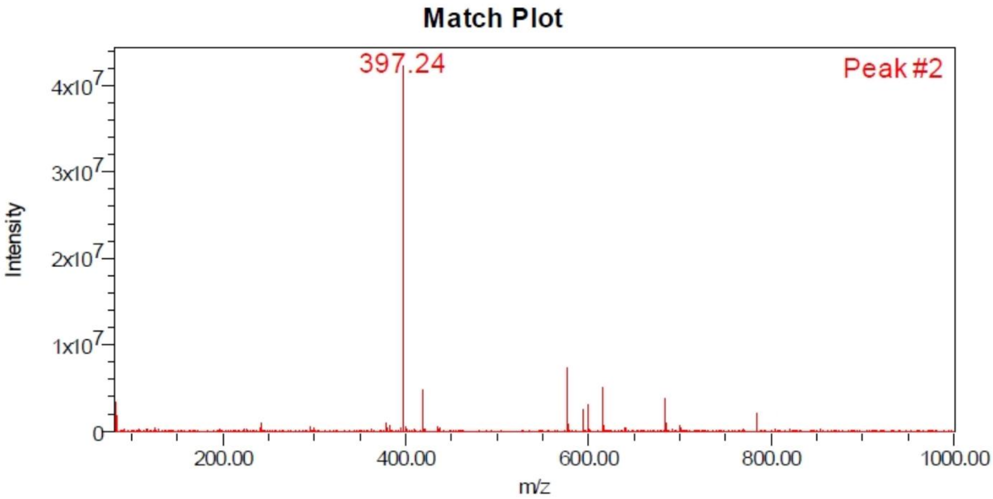

Figure S2. MS (used techniques) spectrum of **3n**

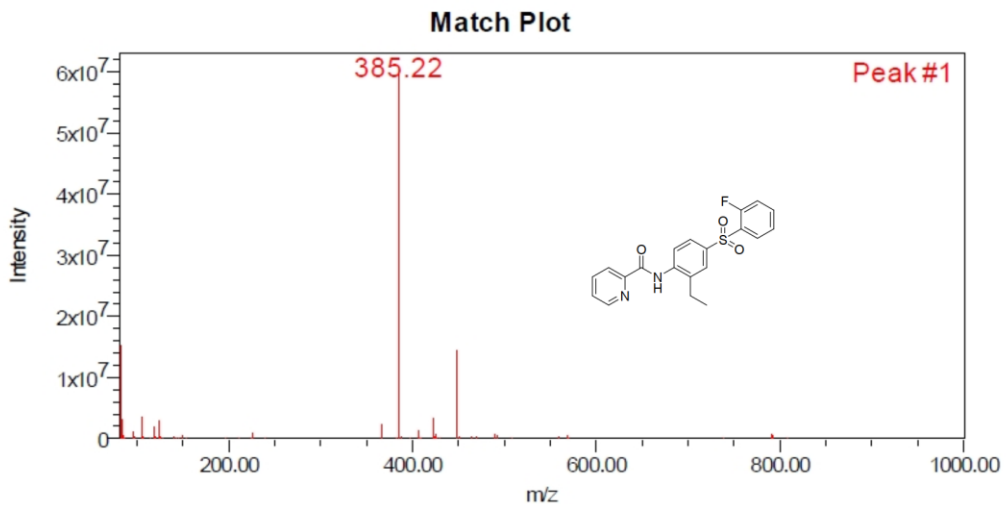

Figure S3. MS (used techniques) spectrum of **3o**

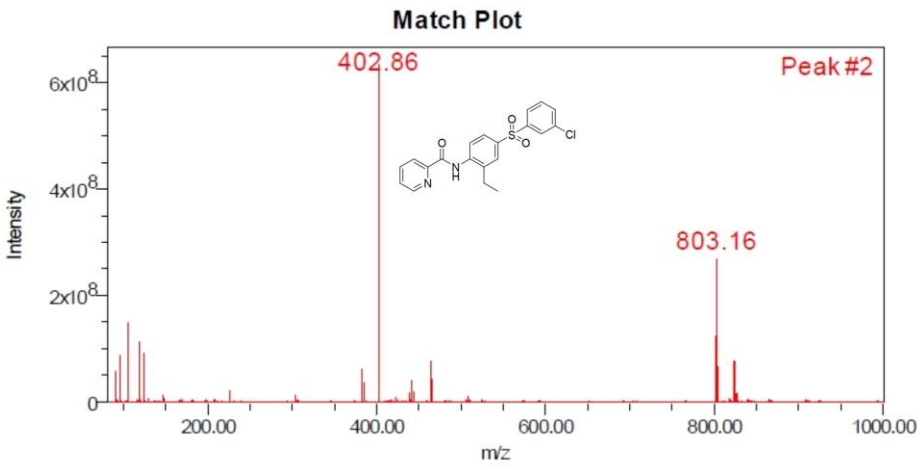

Figure S4. MS (used techniques) spectrum of **3p**

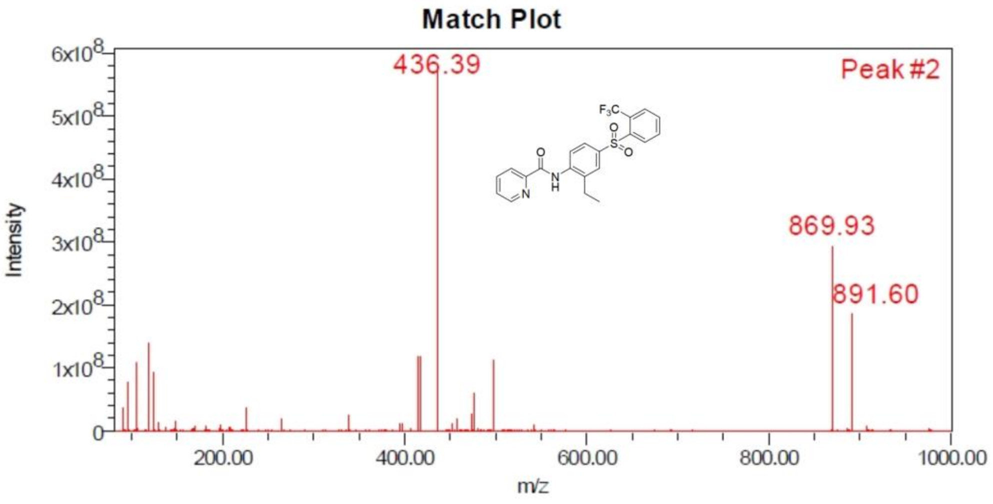

Figure S5. MS (used techniques) spectrum of **3q**

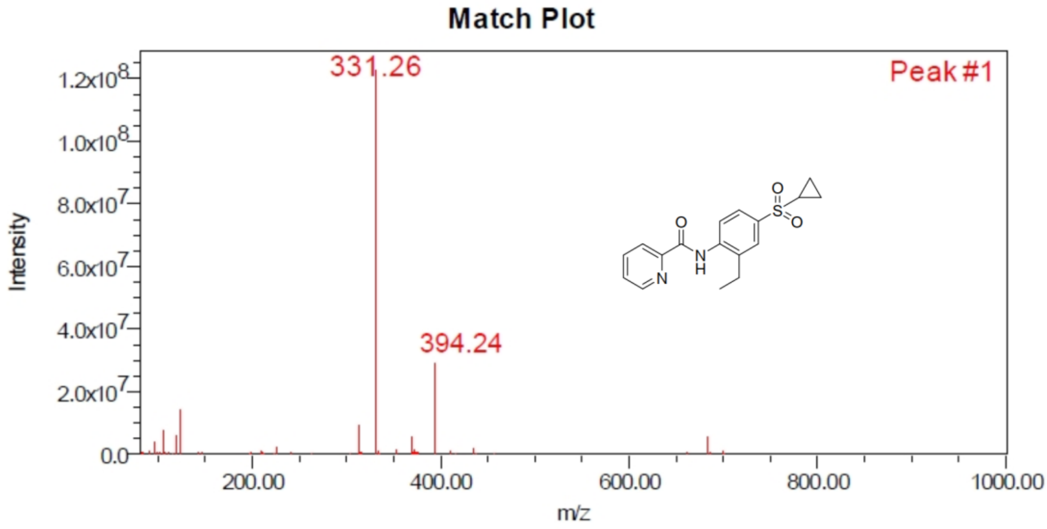

Figure S6. MS (used techniques) spectrum of **3z**

6. ^{1}H and ^{13}C NMR spectra of these compounds

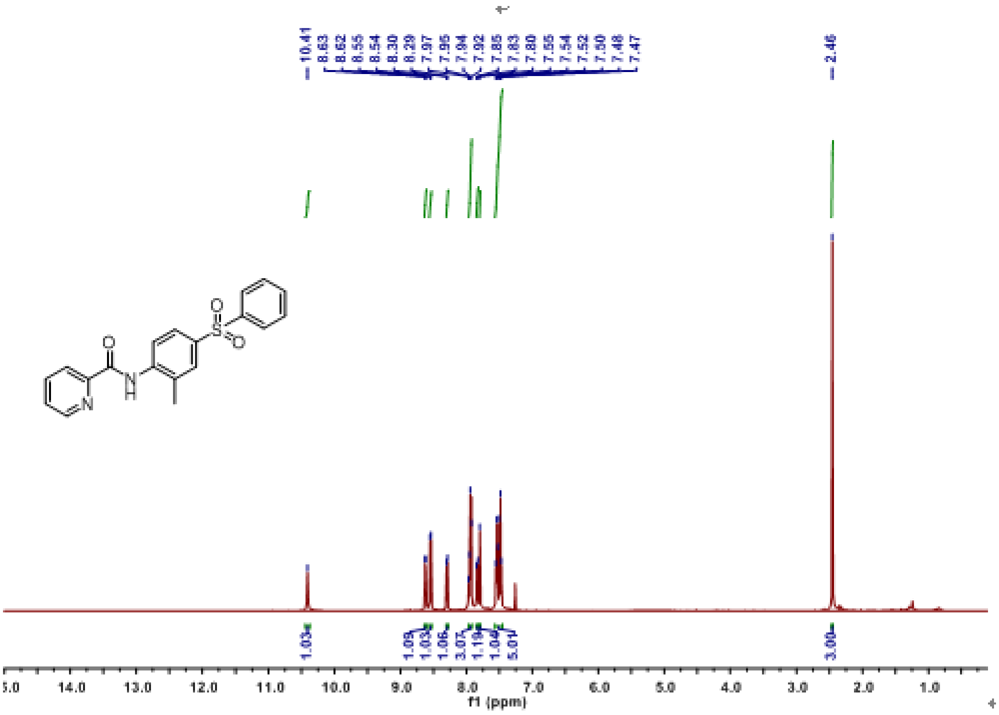

^{1}**H NMR** Spectrum of compound **3a**

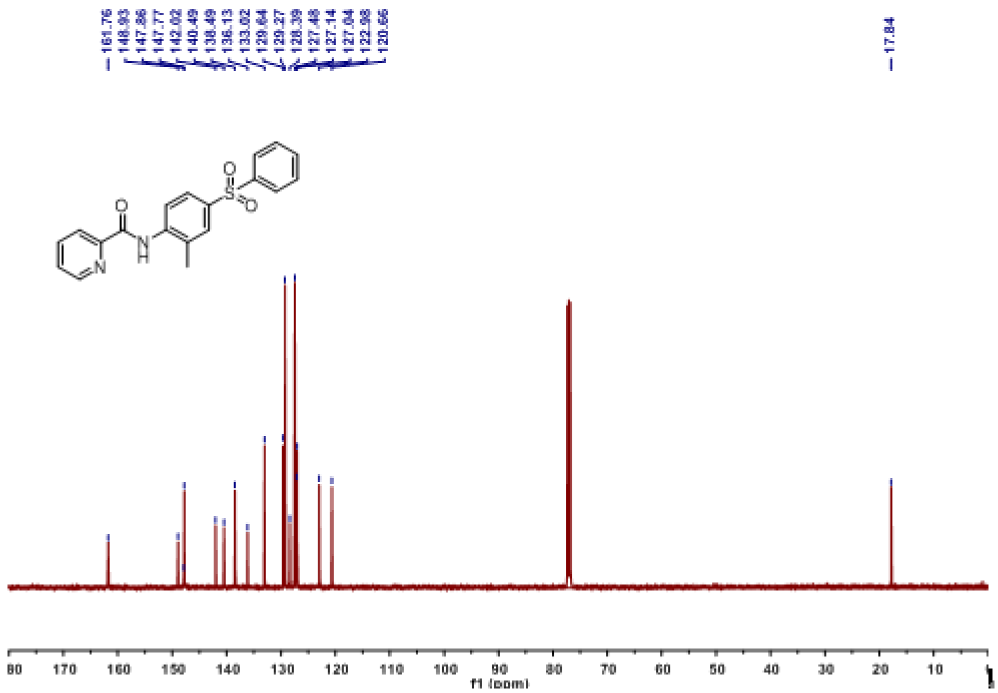

^{13}C **NMR** Spectrum of compound **3a**

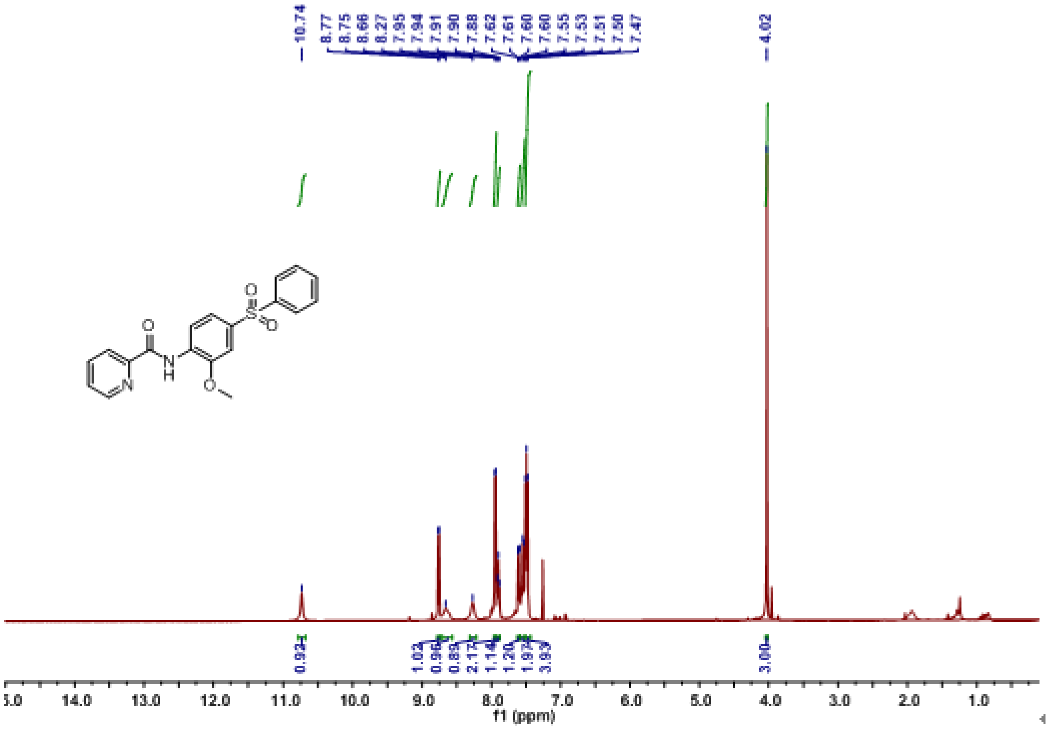

^{1}H **NMR** Spectrum of compound **3b**

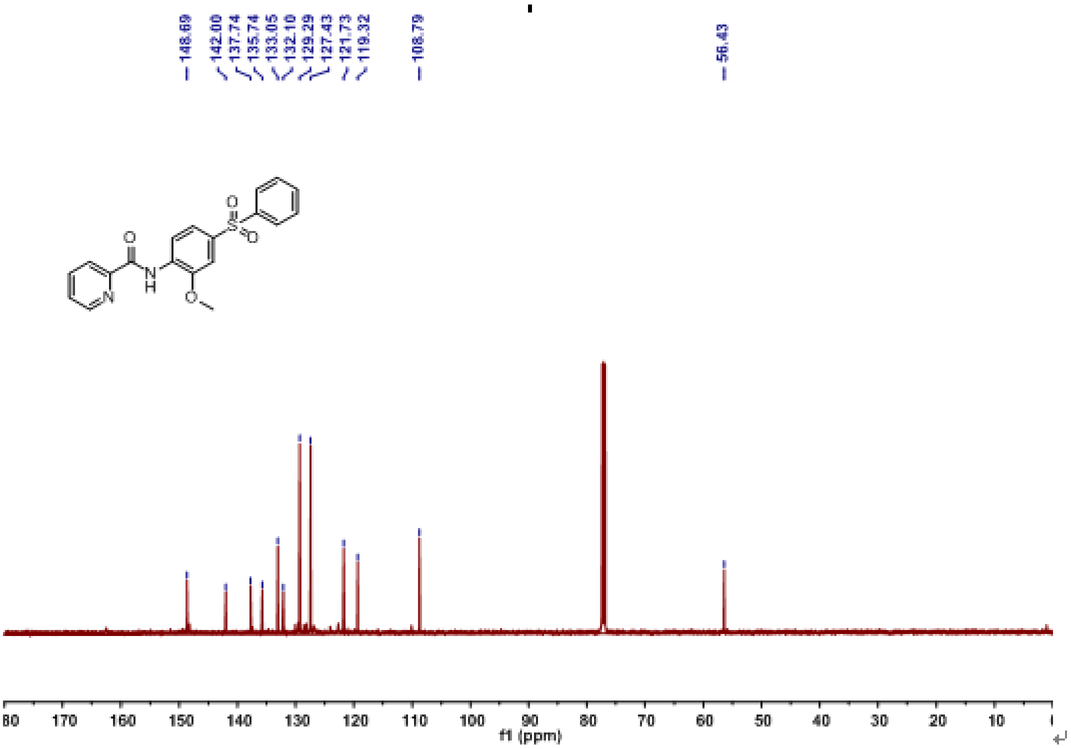

^{13}C **NMR** Spectrum of compound **3b**

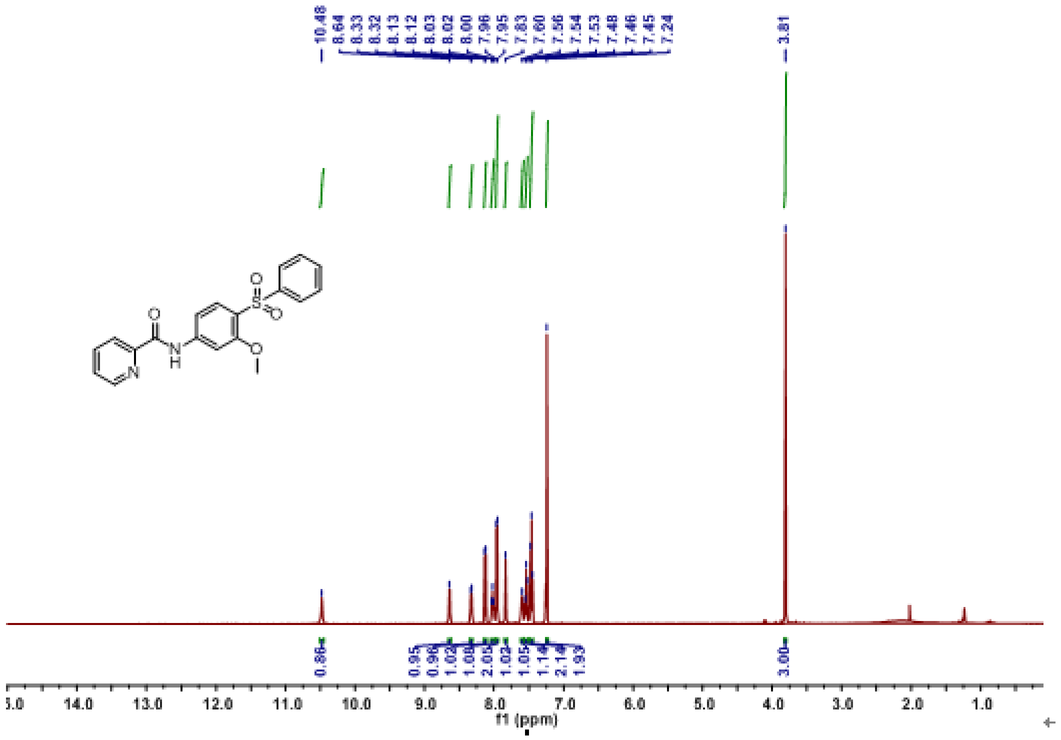

^{1}H **NMR** Spectrum of compound **3c**

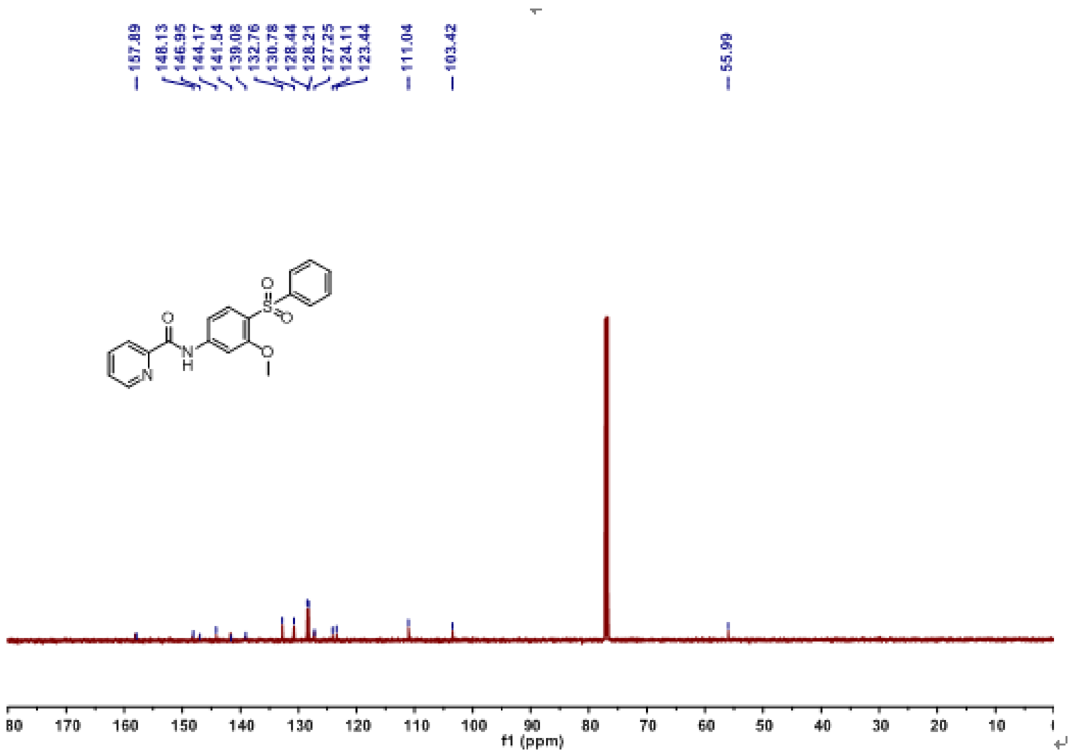

^{13}**C NMR** Spectrum of compound **3c**

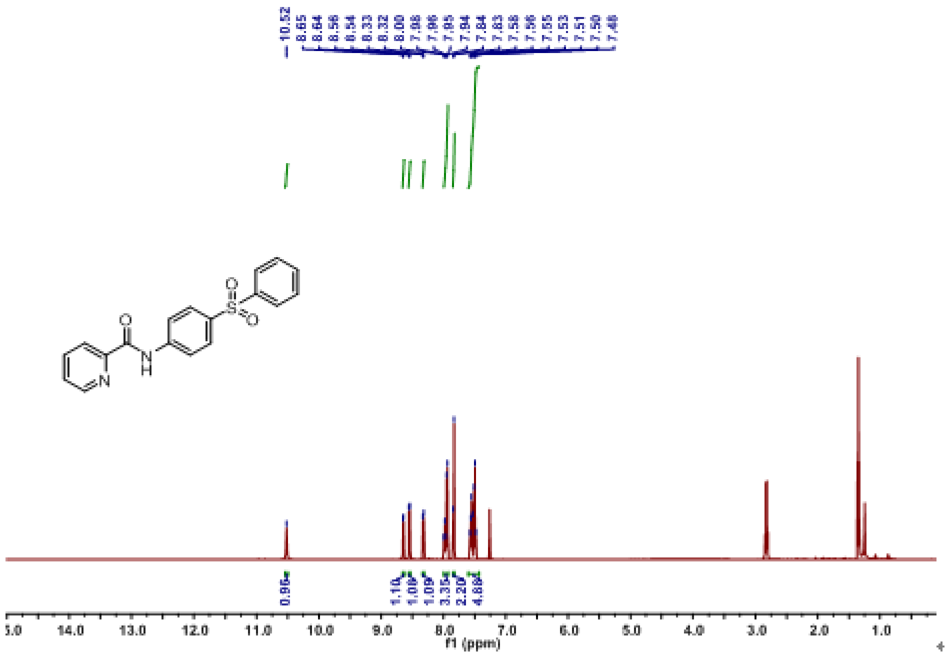

^{1}**H NMR** Spectrum of compound **3d**

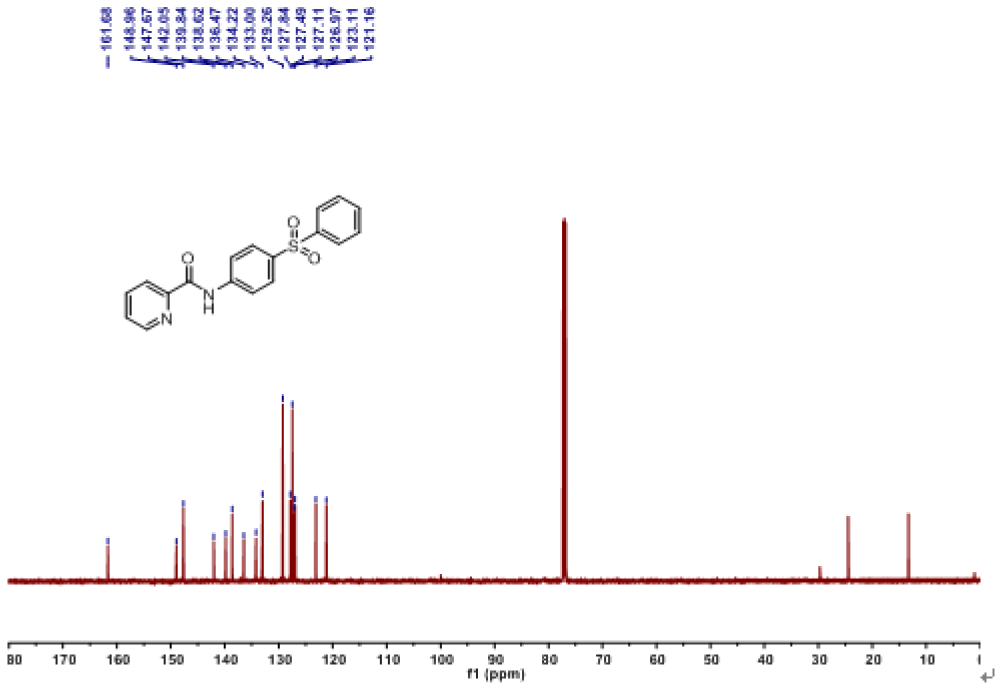

^{13}C NMR Spectrum of compound **3d**

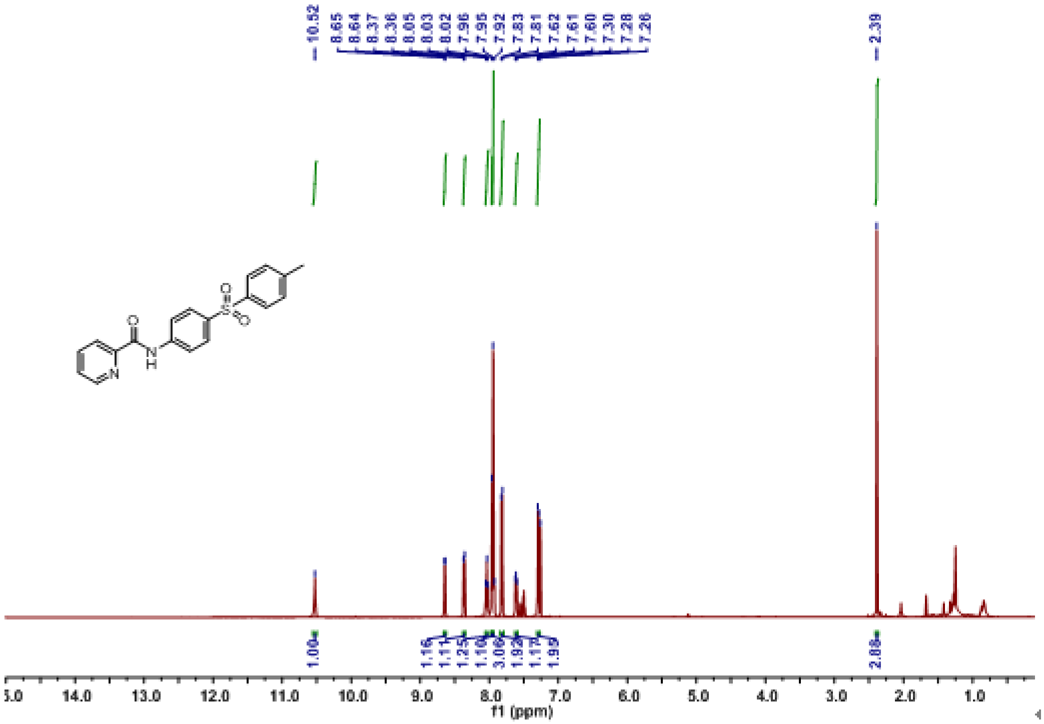

^{1}H NMR Spectrum of compound **3e**

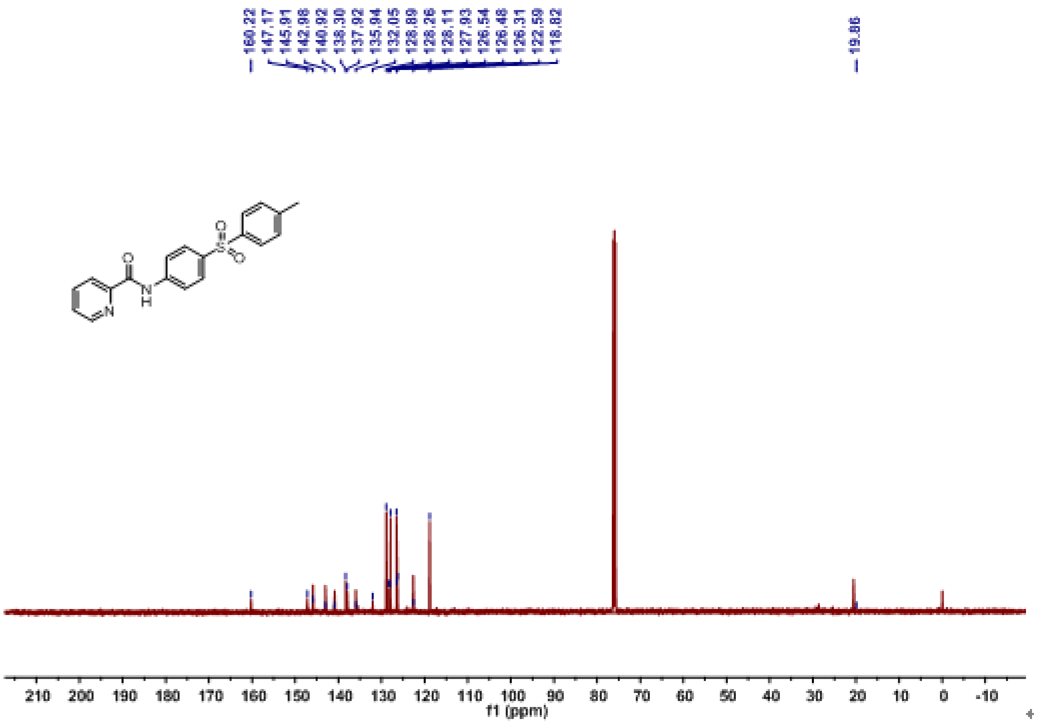

^{13}C NMR Spectrum of compound **3e**

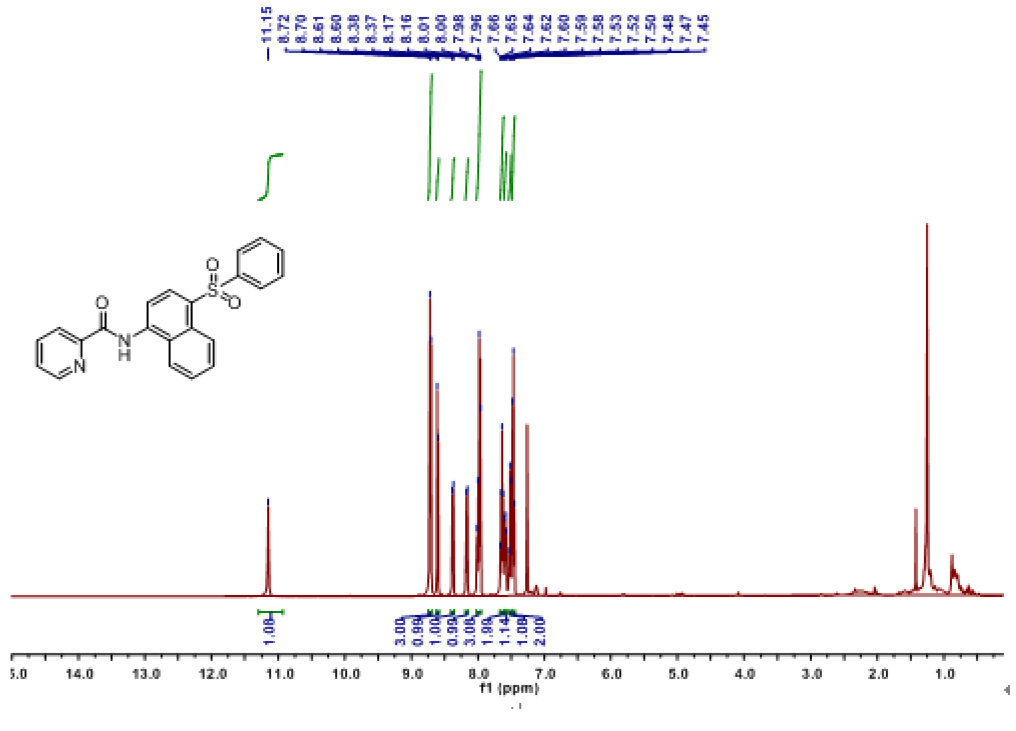

^{1}H NMR Spectrum of compound **3h**

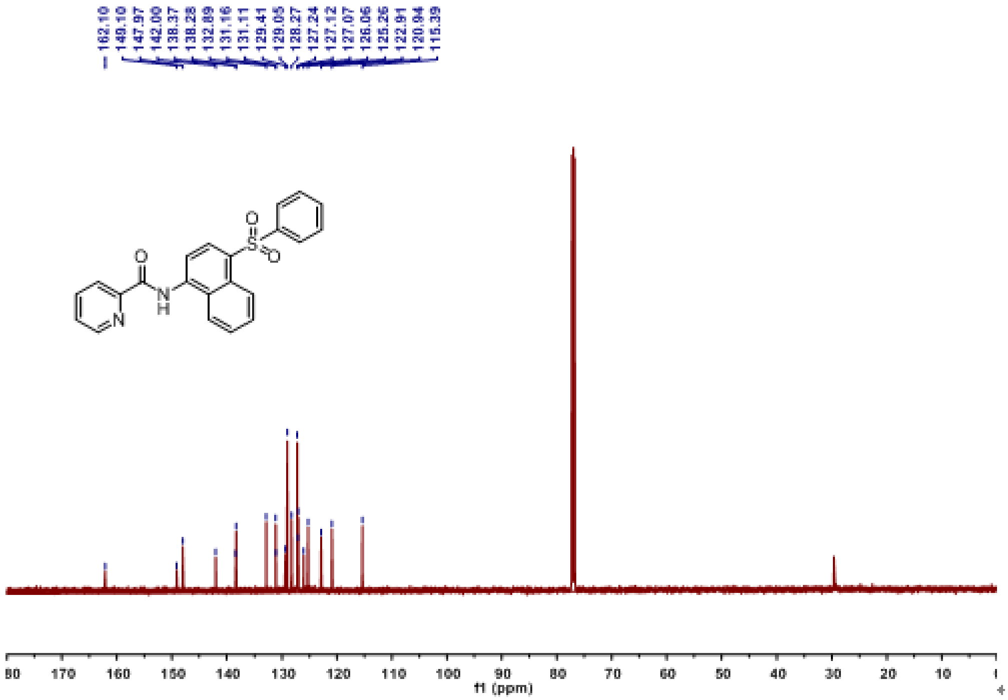

^{13}**C NMR** Spectrum of compound **3h**

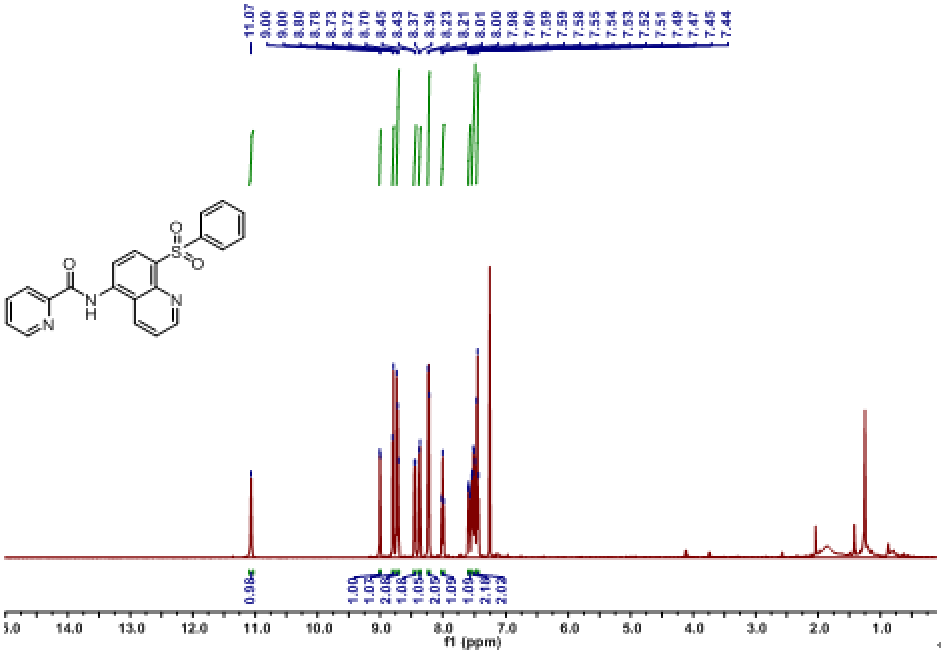

^{1}**H NMR** Spectrum of compound **3i**

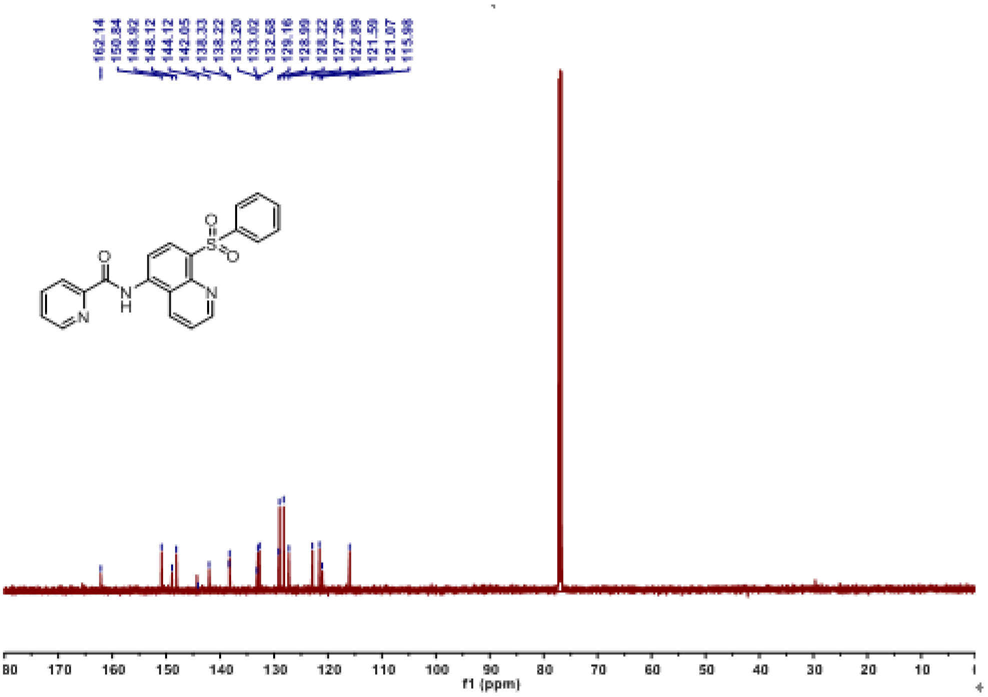

^{13}C **NMR** Spectrum of compound **3i**

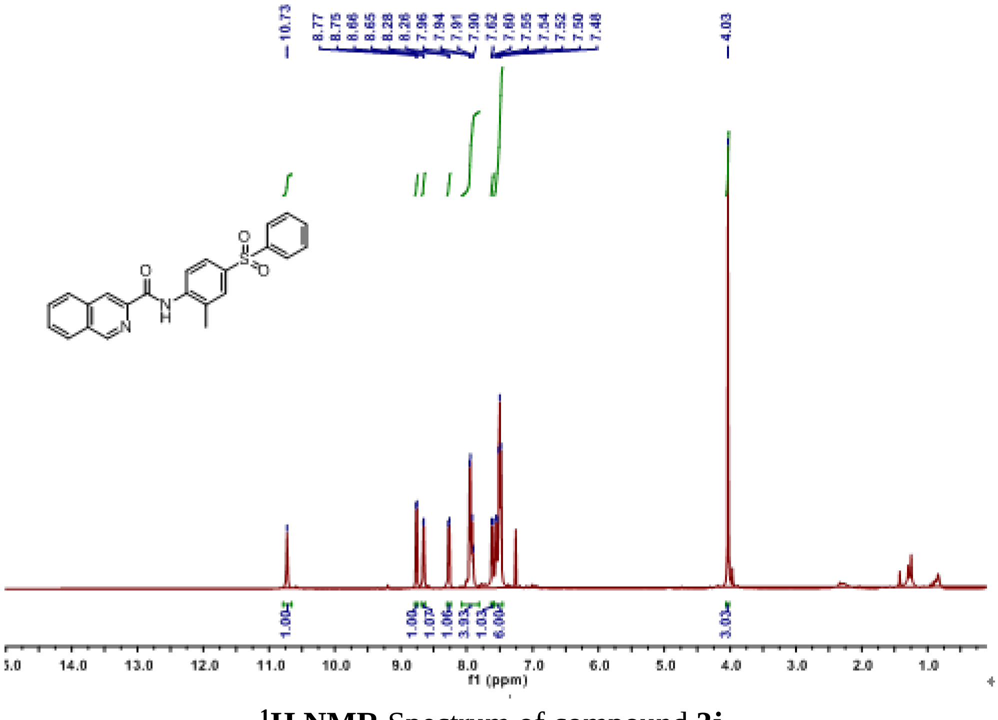

^{1}H **NMR** Spectrum of compound **3j**

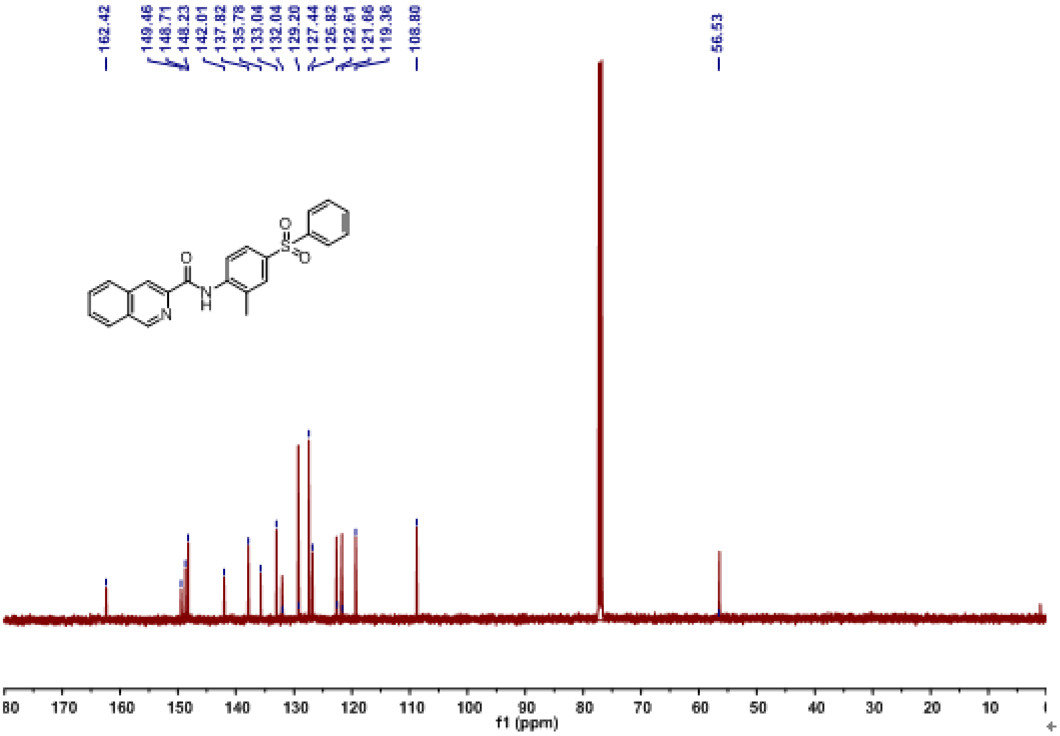

^{13}C NMR Spectrum of compound **3j**

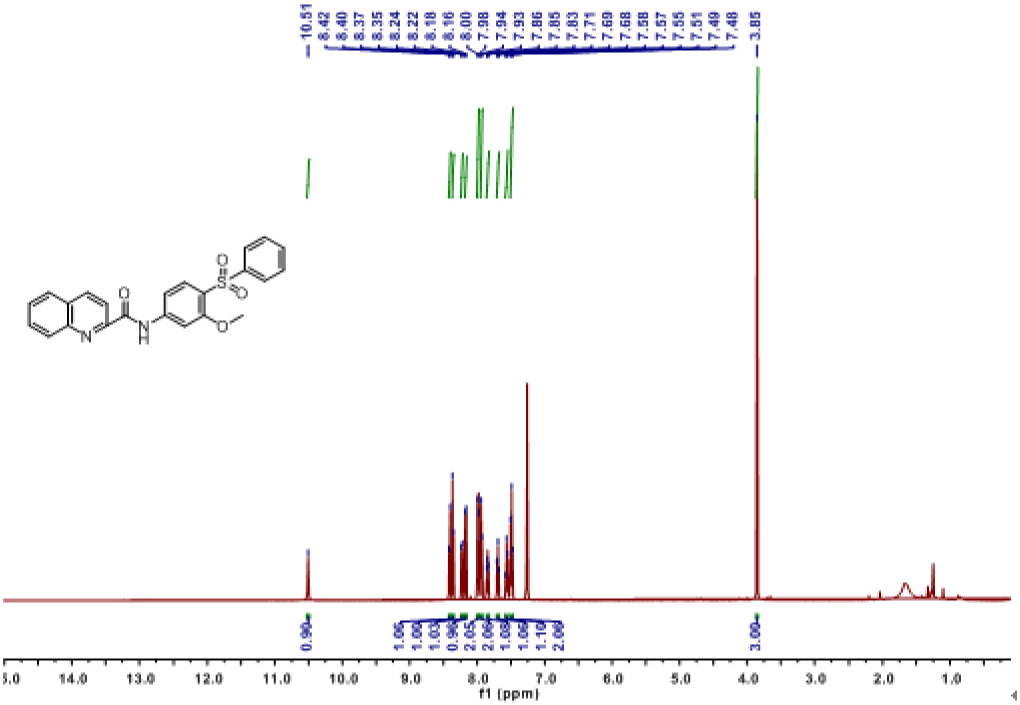

^{1}H NMR Spectrum of compound **3k**

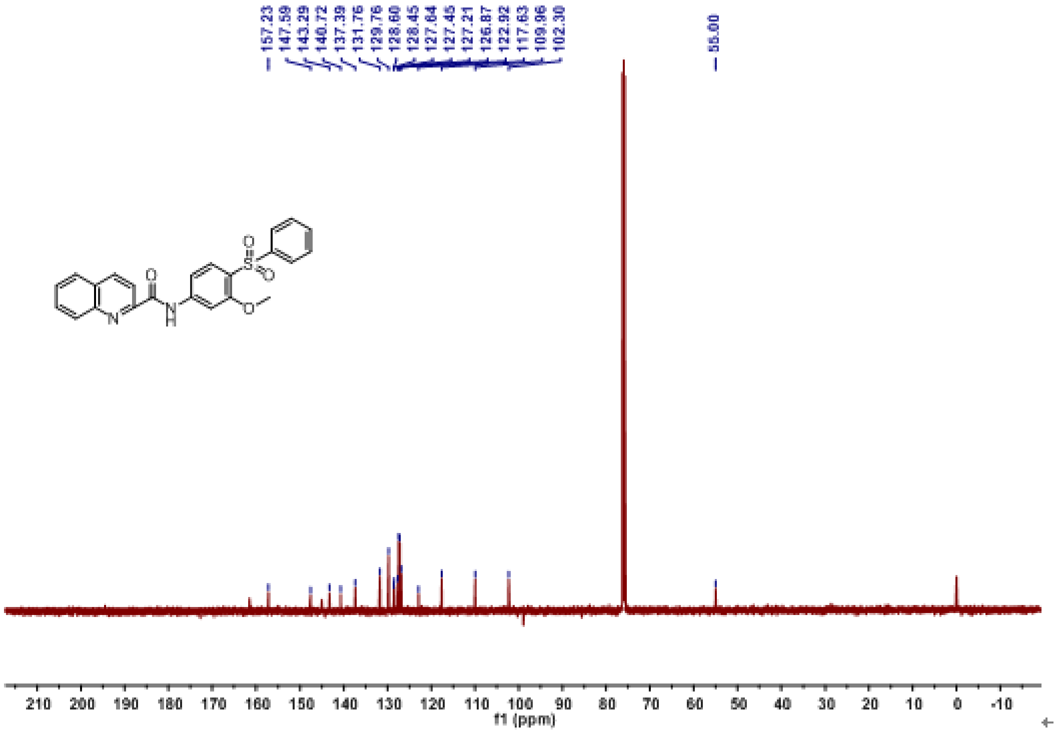

^{13}C NMR Spectrum of compound **3k**

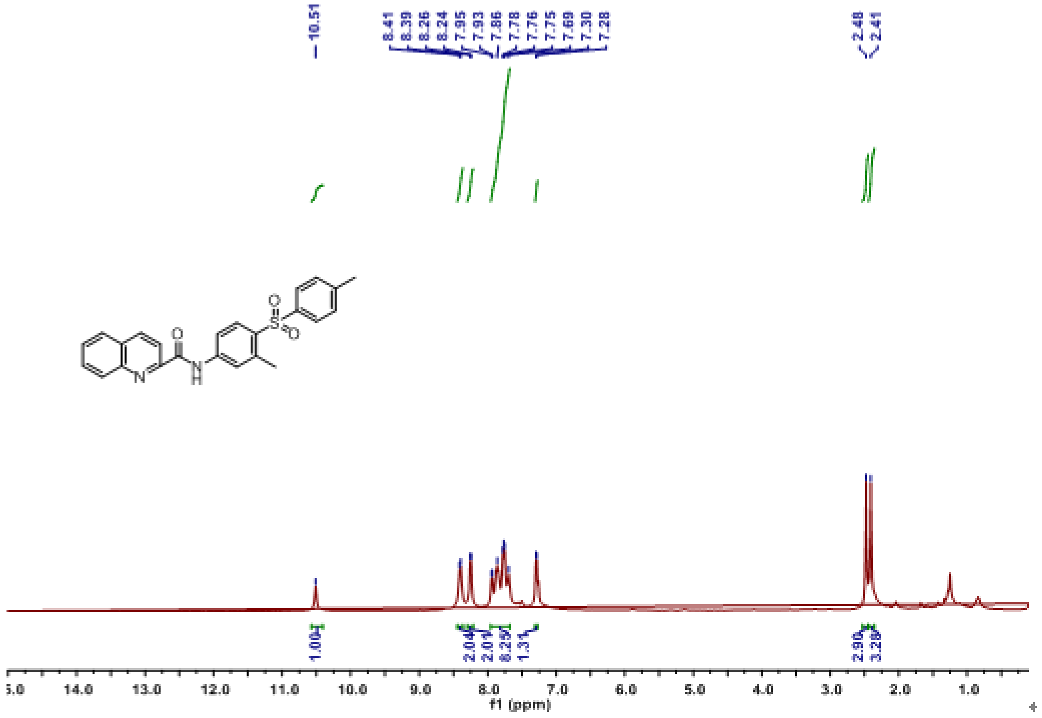

^{1}H NMR Spectrum of compound **3l**

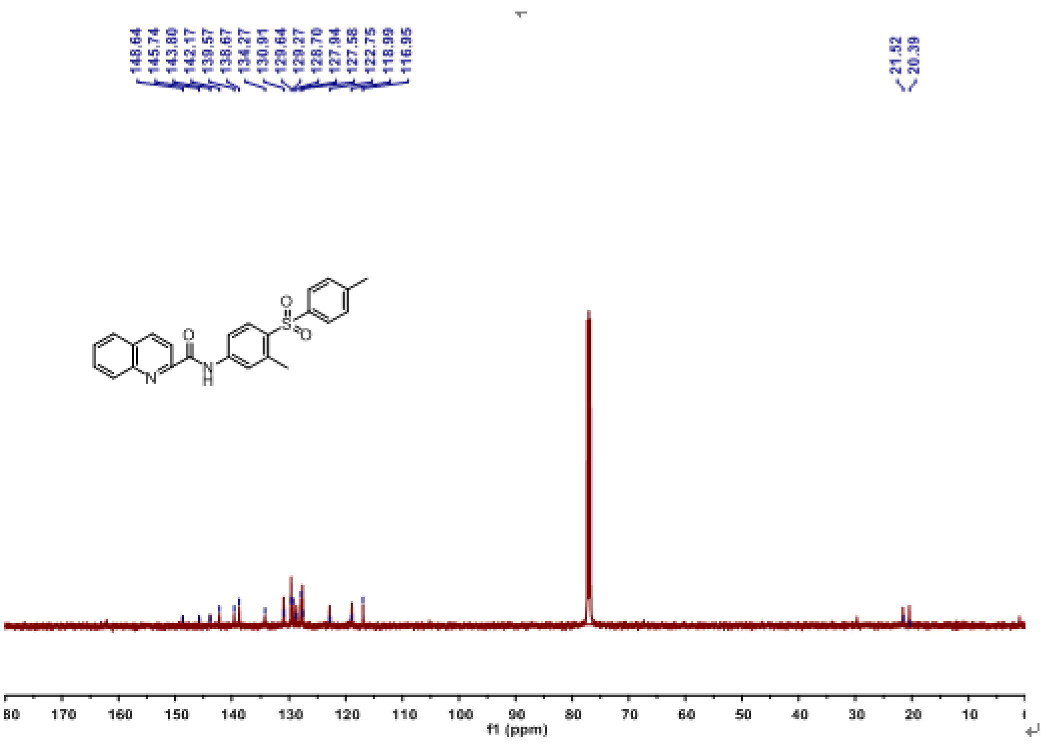

^{13}C **NMR** Spectrum of compound **3l**

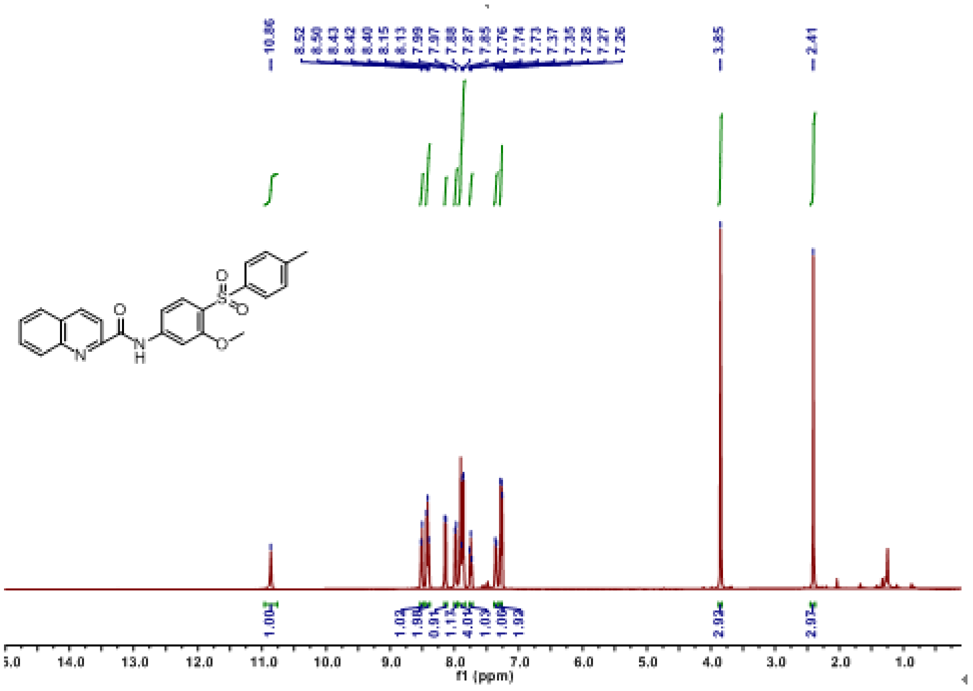

^{1}H **NMR** Spectrum of compound **3m**

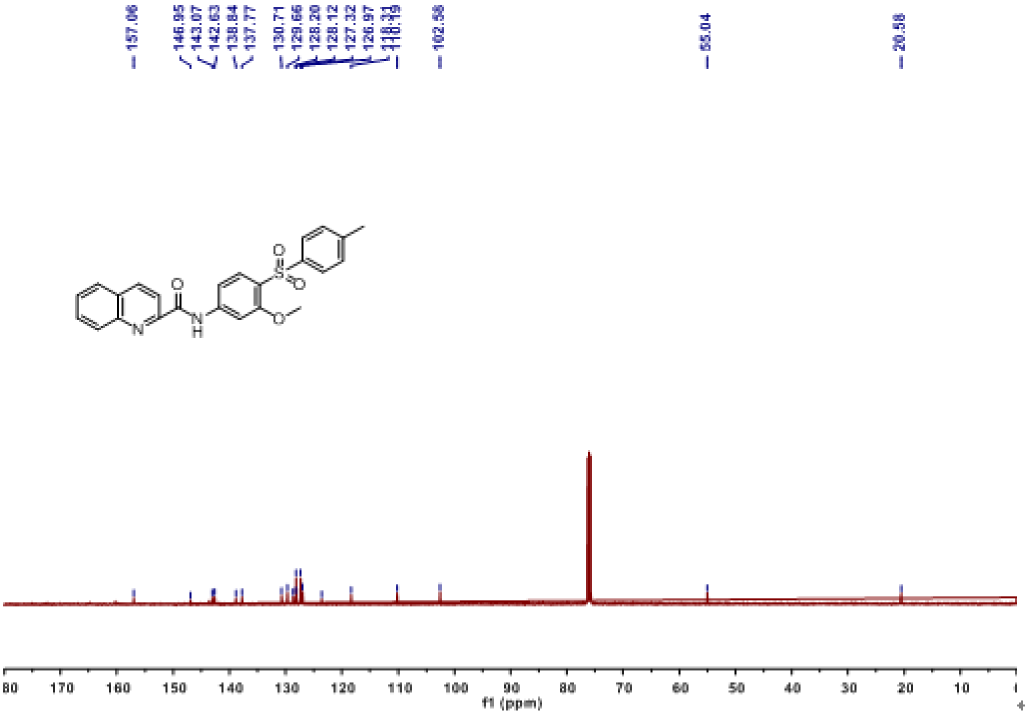

^{13}C **NMR** Spectrum of compound **3m**

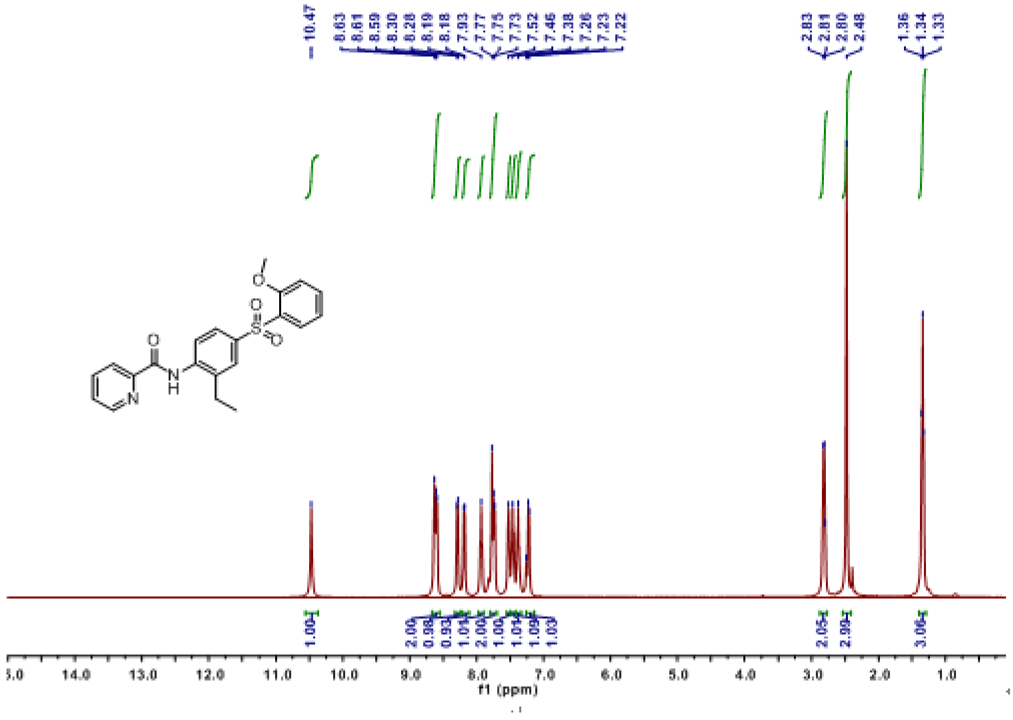

^{1}H **NMR** Spectrum of compound **3n**

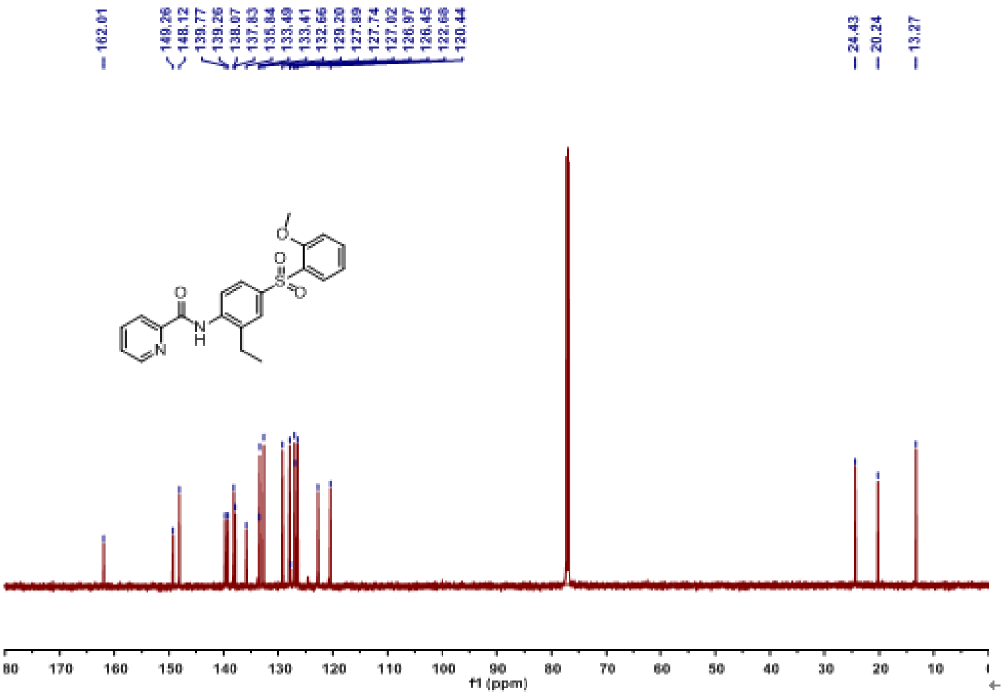

^{13}C NMR Spectrum of compound **3n**

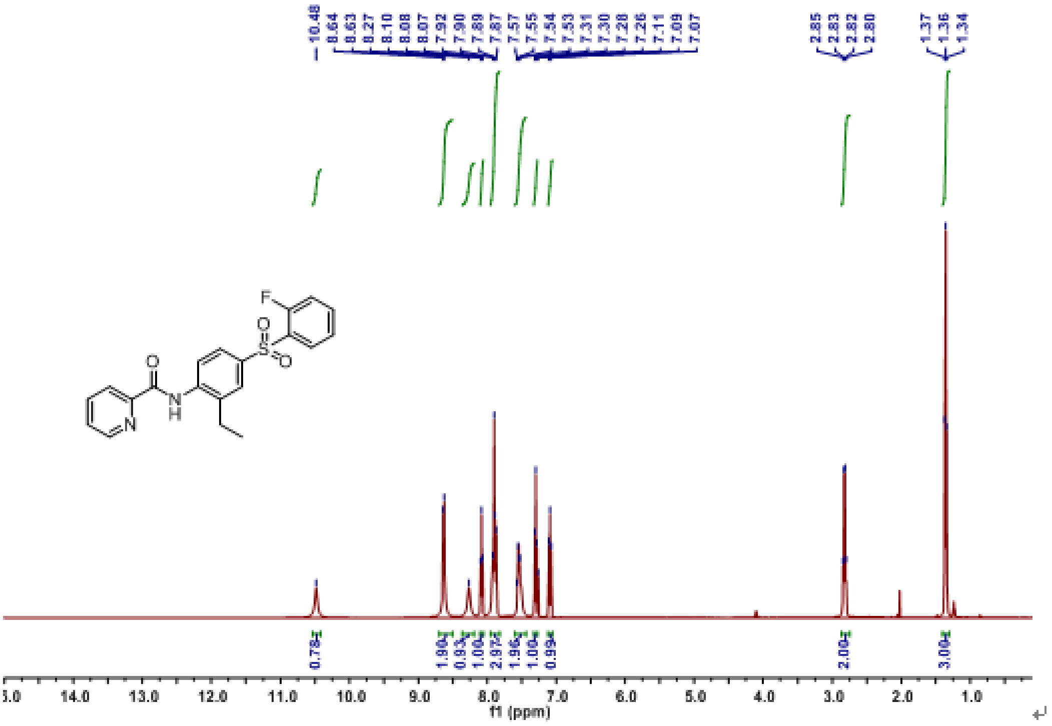

^{1}H NMR Spectrum of compound **3o**

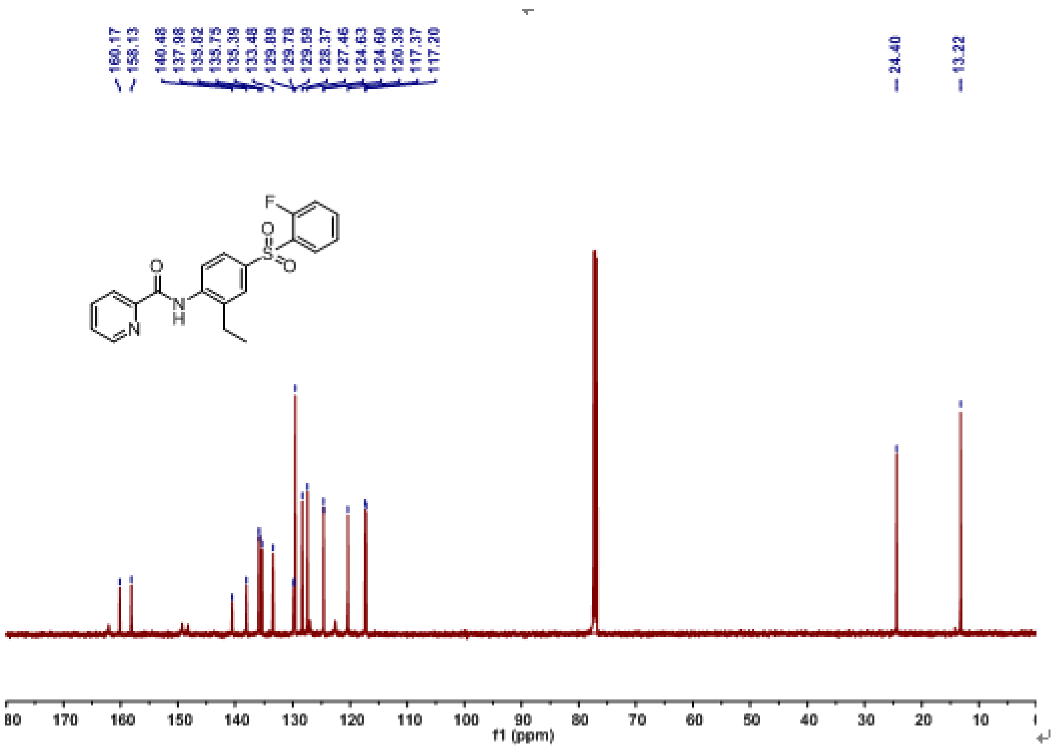

^{13}C NMR Spectrum of compound **3o**

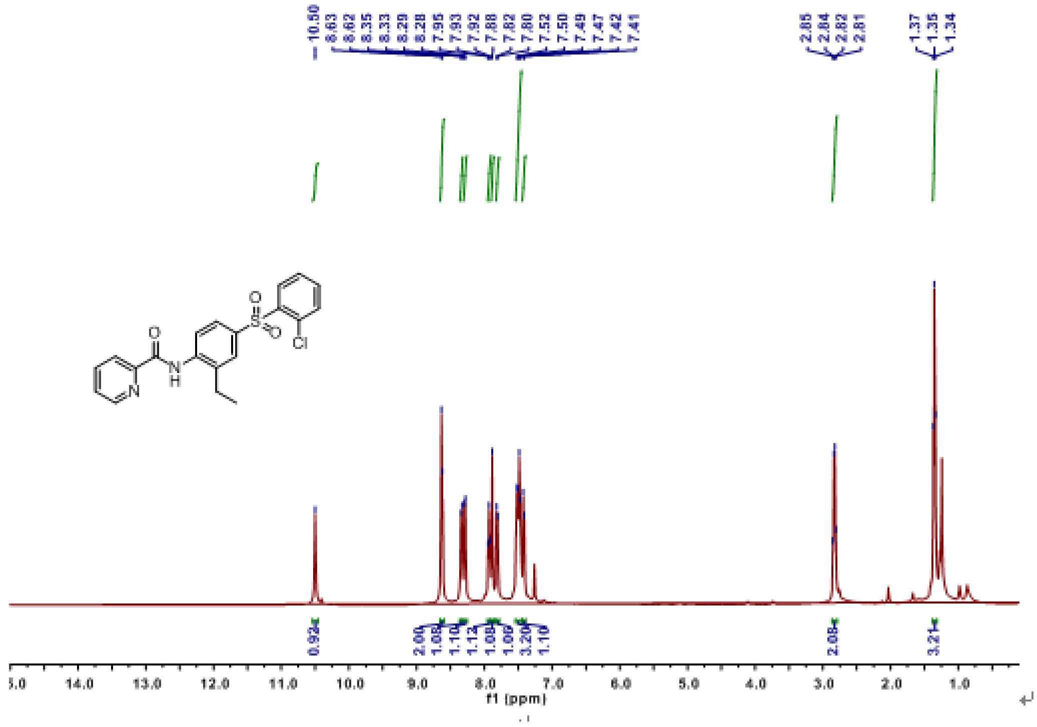

^{1}H NMR Spectrum of compound **3p**

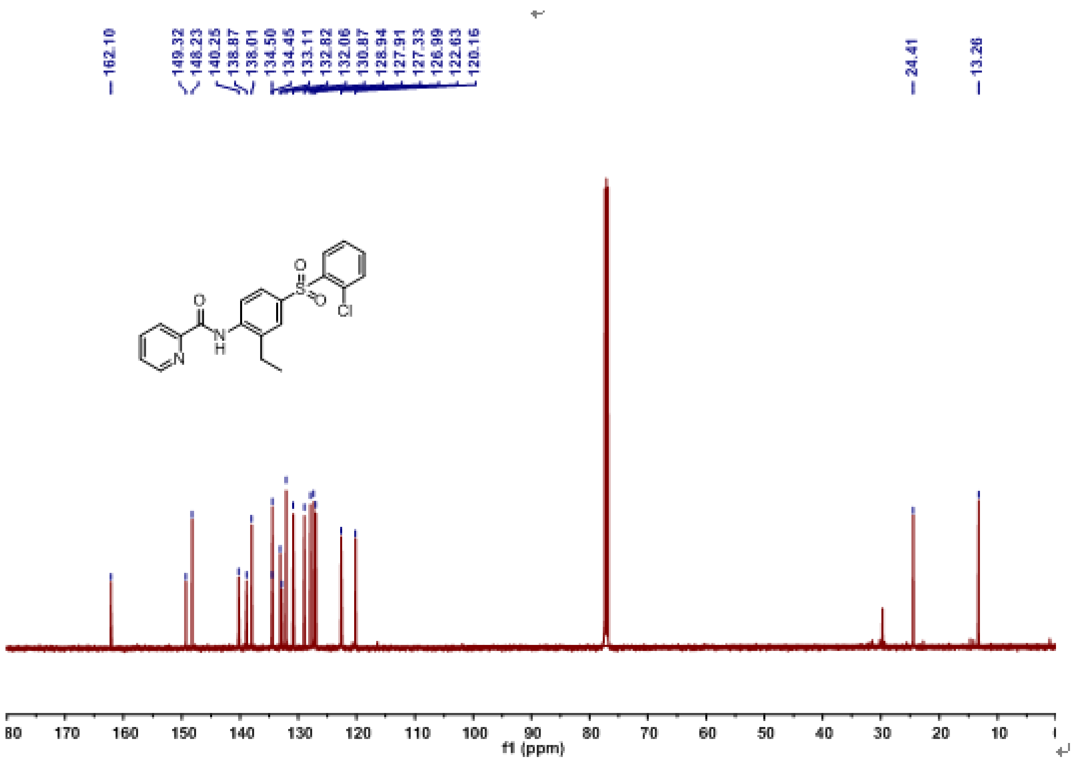

^{13}**C NMR** Spectrum of compound **3p**

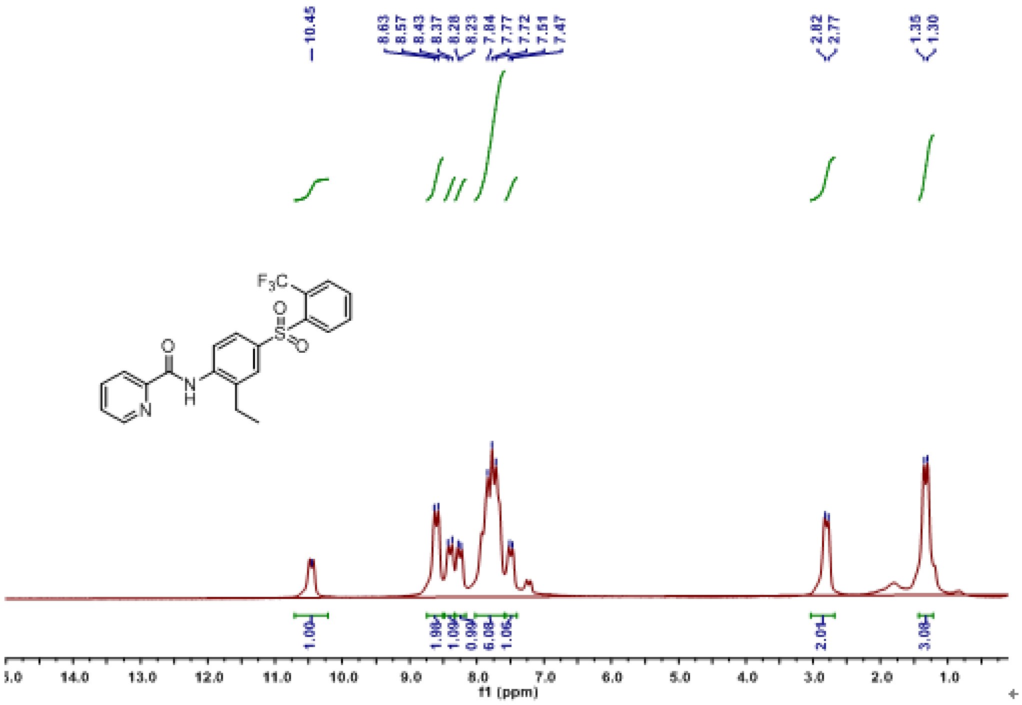

^{1}**H NMR** Spectrum of compound **3q**

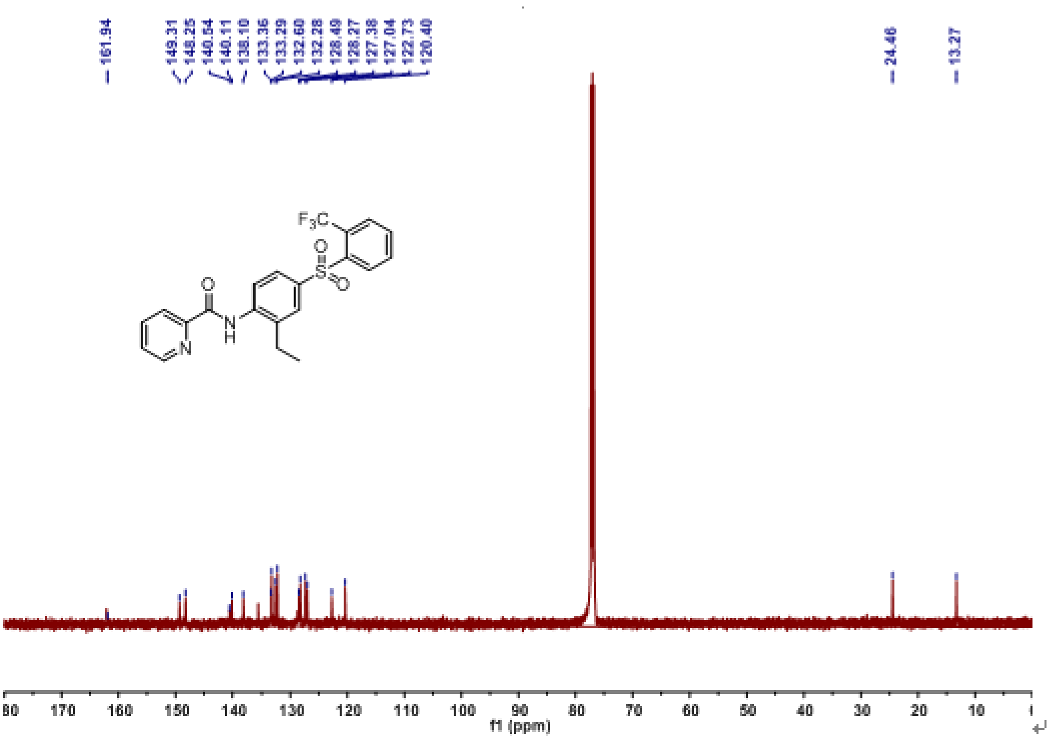

^{13}C NMR Spectrum of compound **3q**

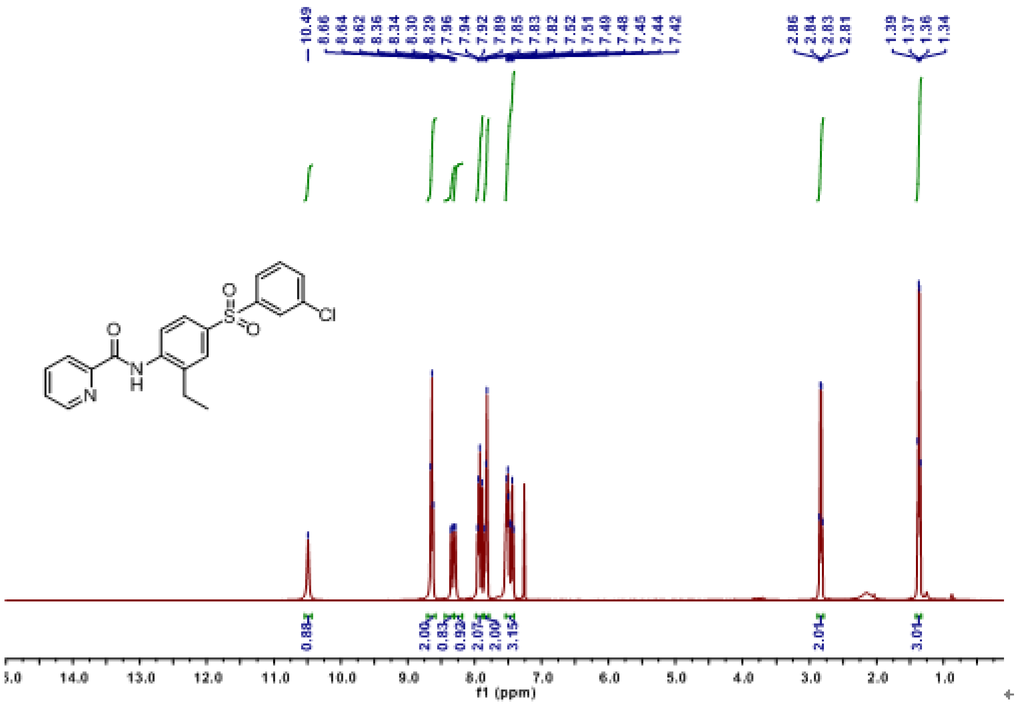

^{1}H NMR Spectrum of compound **3r**

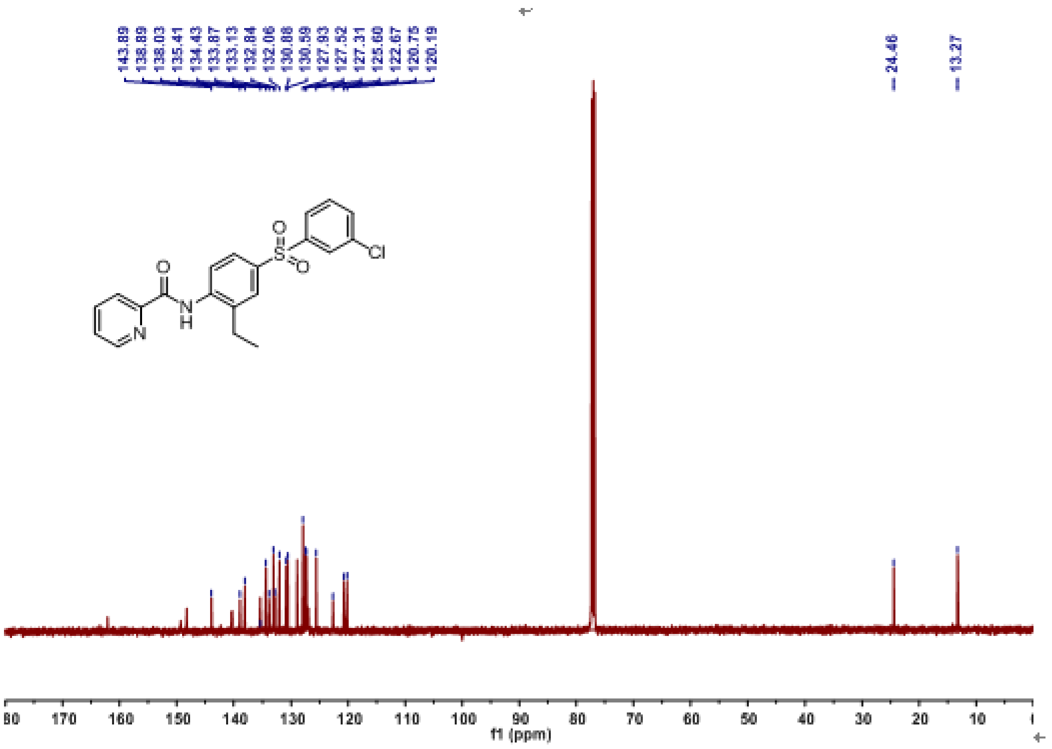

^{13}C NMR Spectrum of compound **3r**

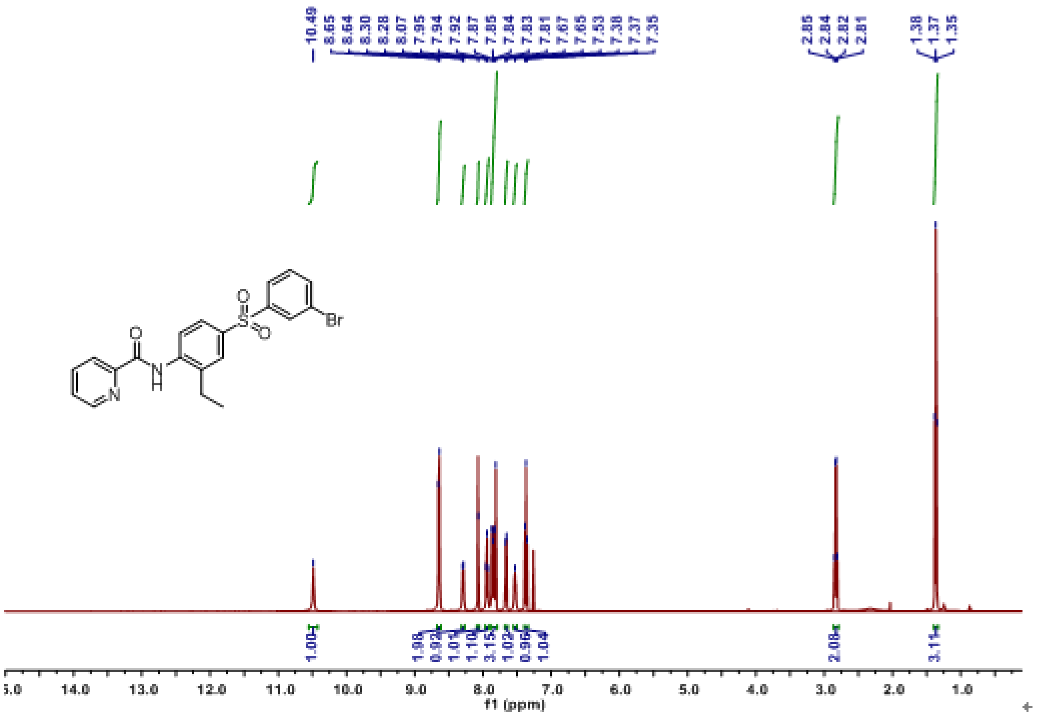

^{1}H NMR Spectrum of compound **3s**

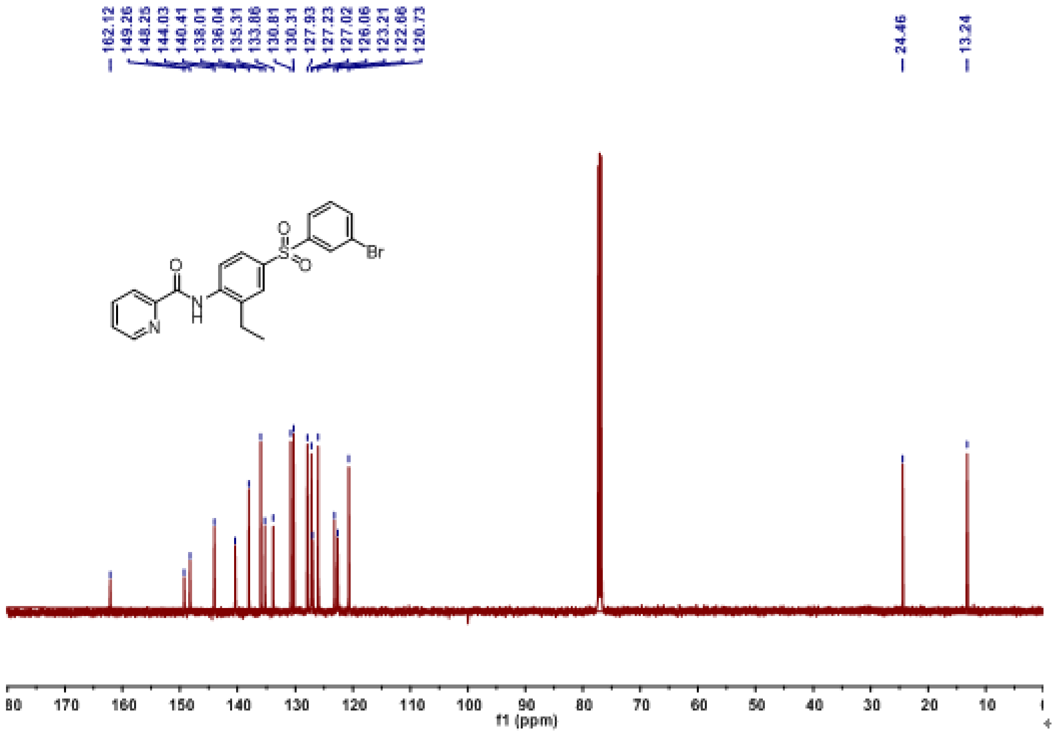

^{13}**C NMR** Spectrum of compound **3s**

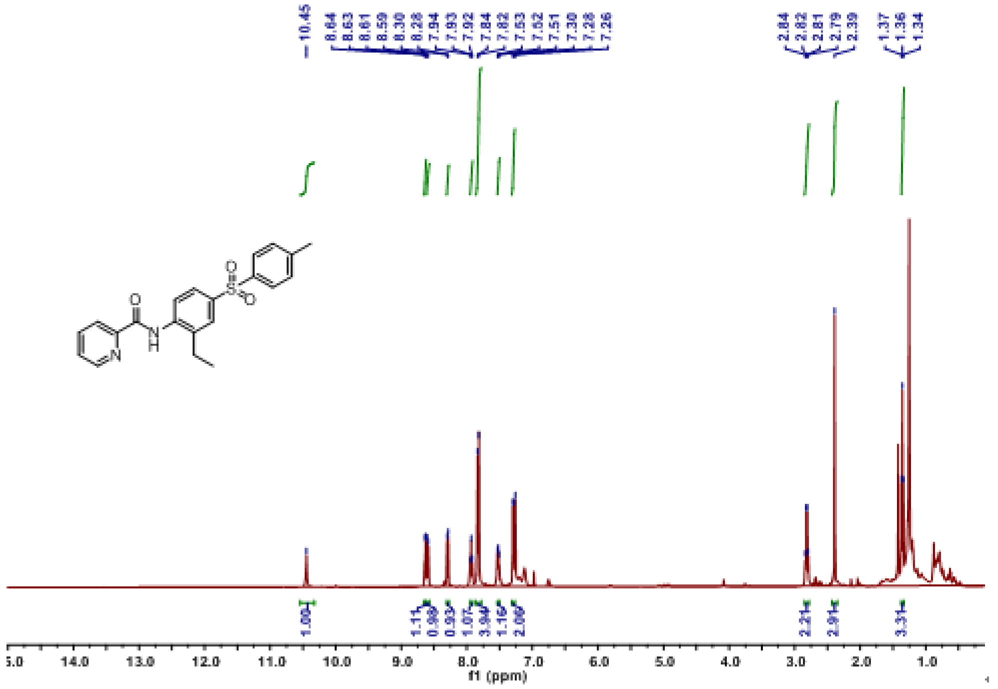

^{1}**H NMR** Spectrum of compound **3t**

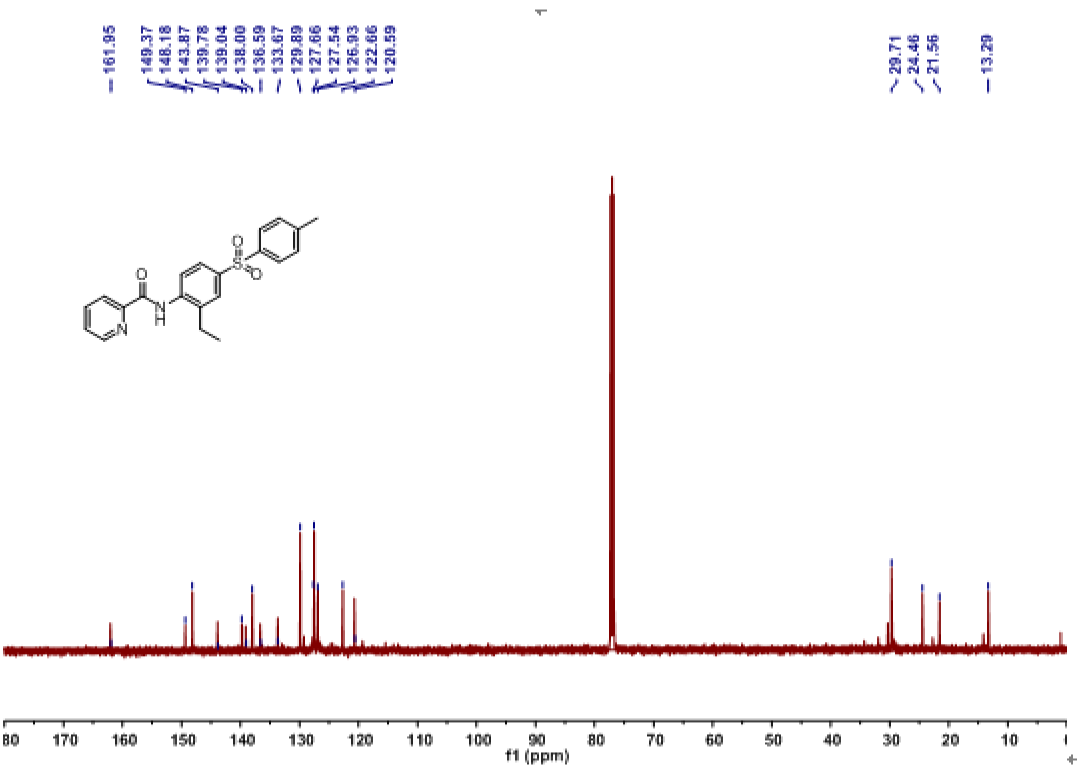

^{13}C NMR Spectrum of compound **3t**

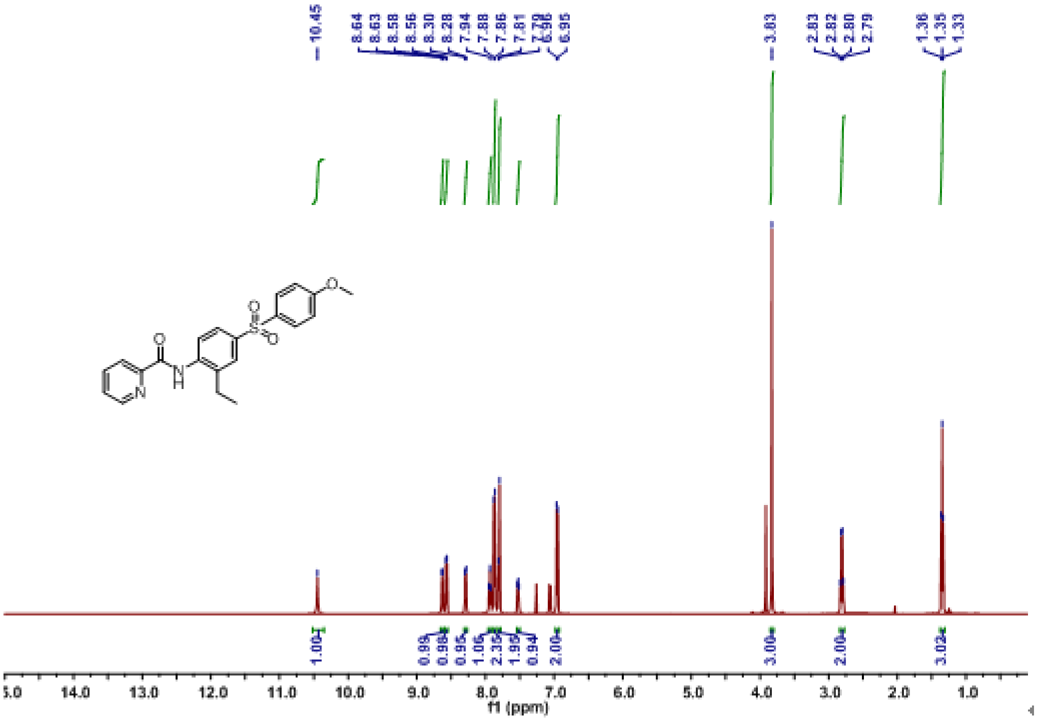

^{1}H NMR Spectrum of compound **3u**

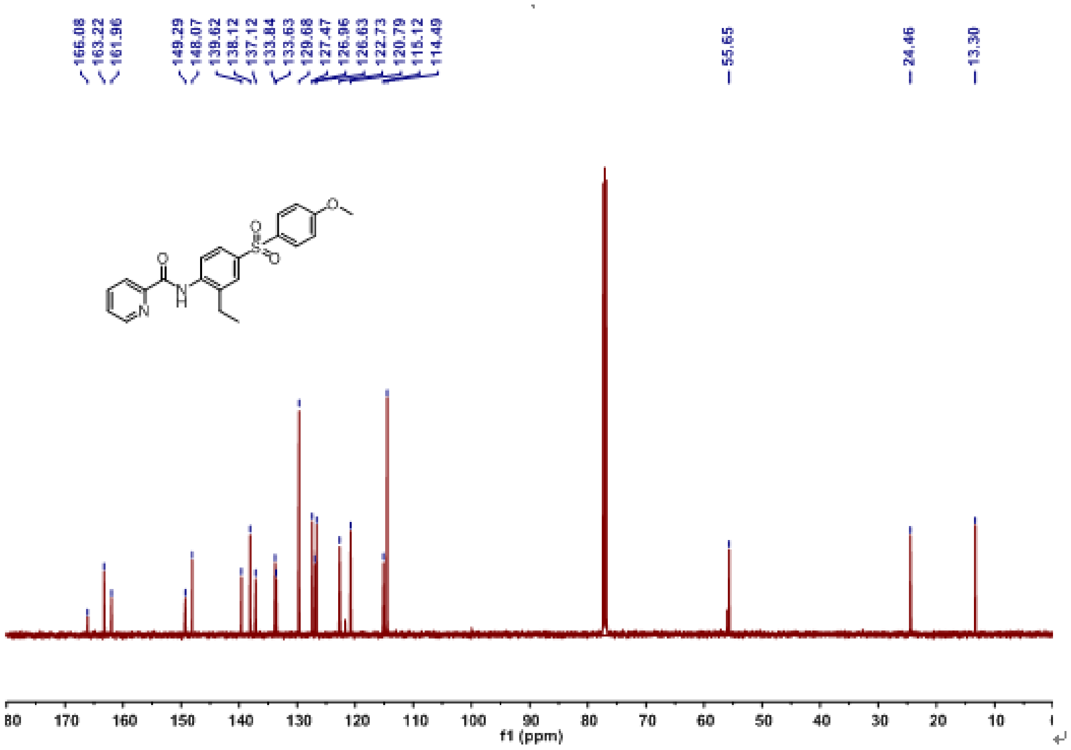

^{13}C **NMR** Spectrum of compound **3u**

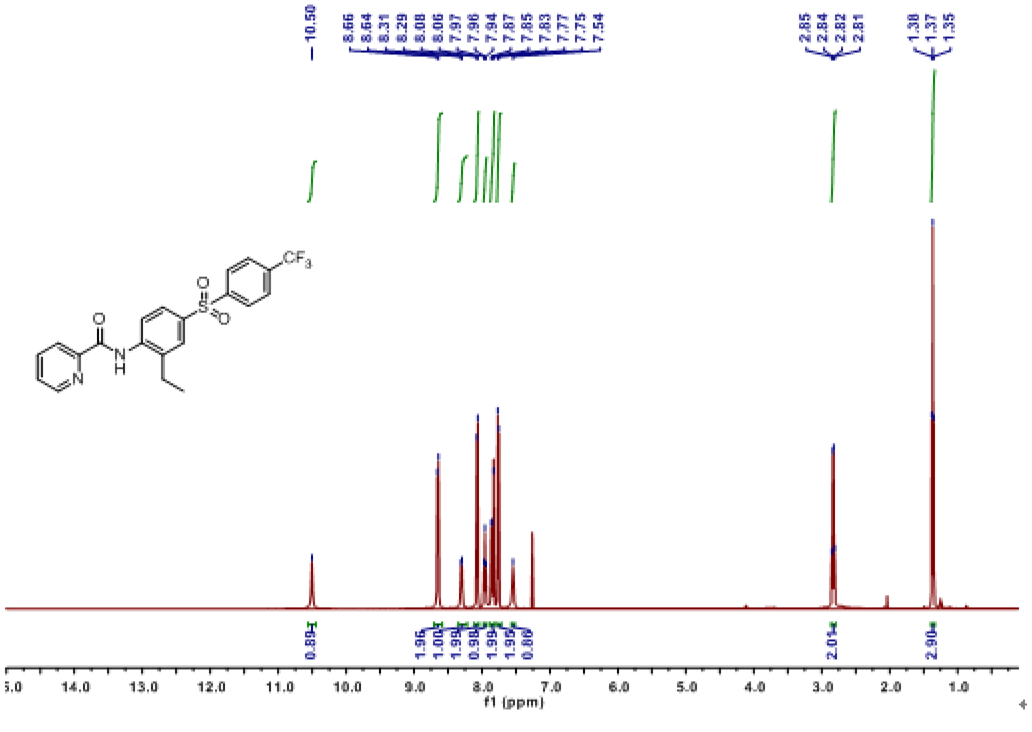

^{1}H **NMR** Spectrum of compound **3v**

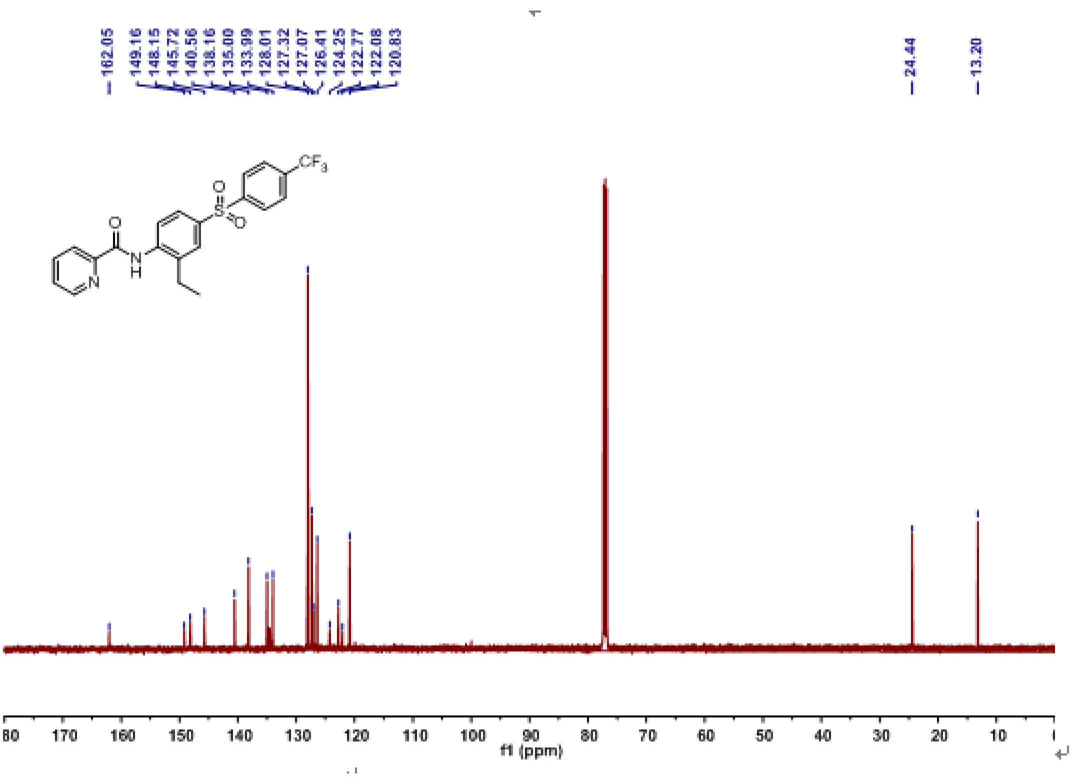

^{13}C NMR Spectrum of compound **3v**

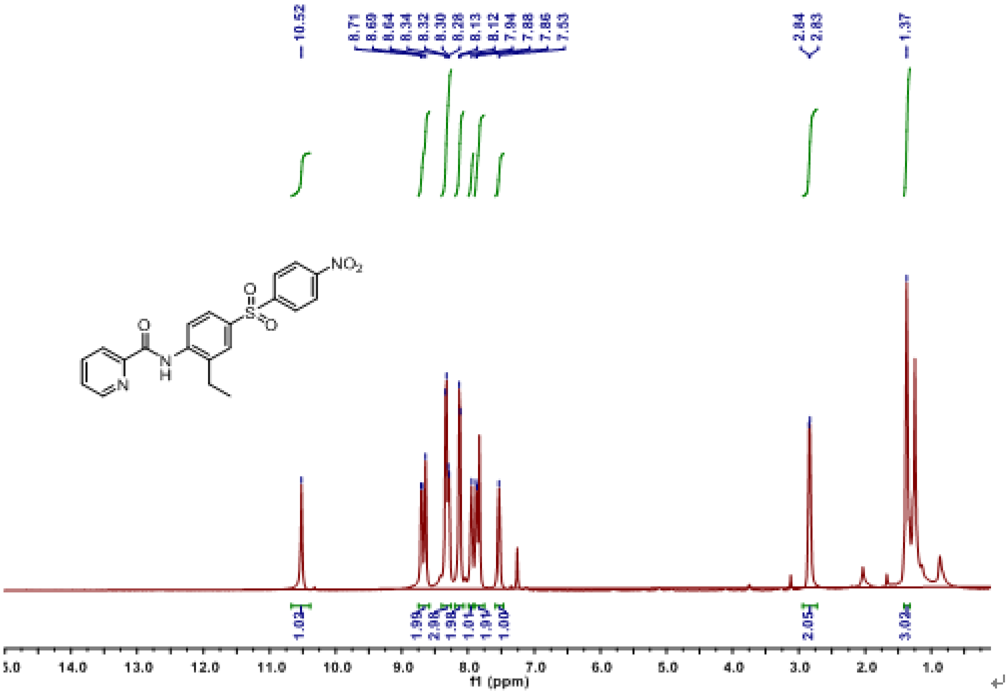

^{1}H NMR Spectrum of compound **3w**

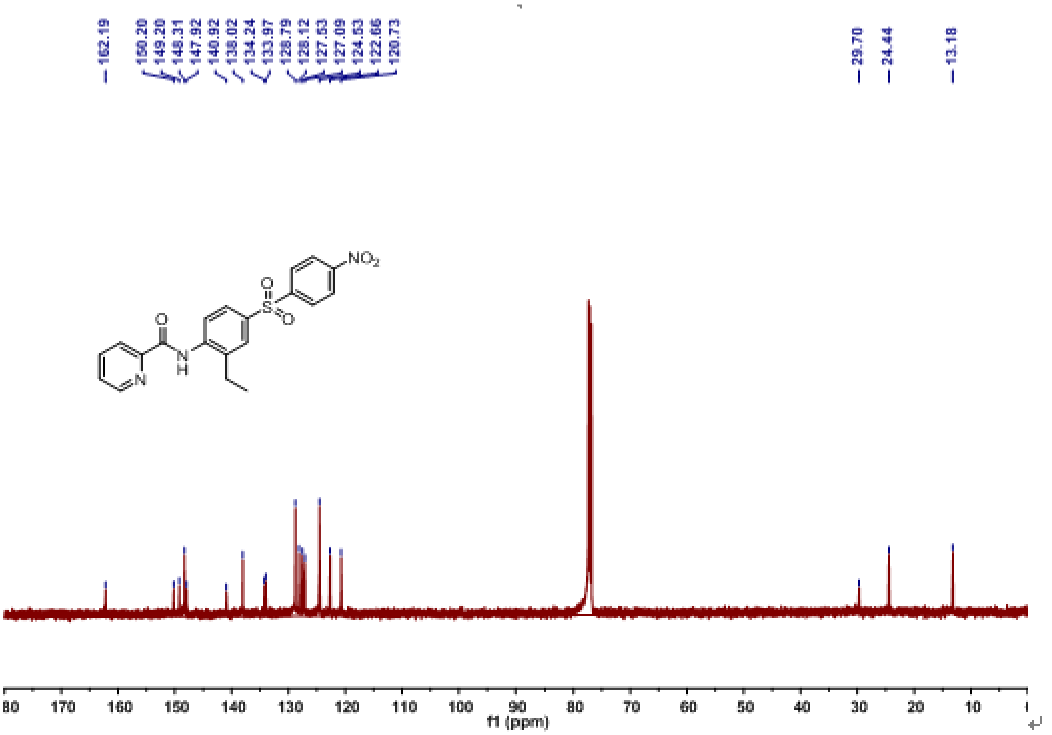

[13] **C NMR** Spectrum of compound **3w**

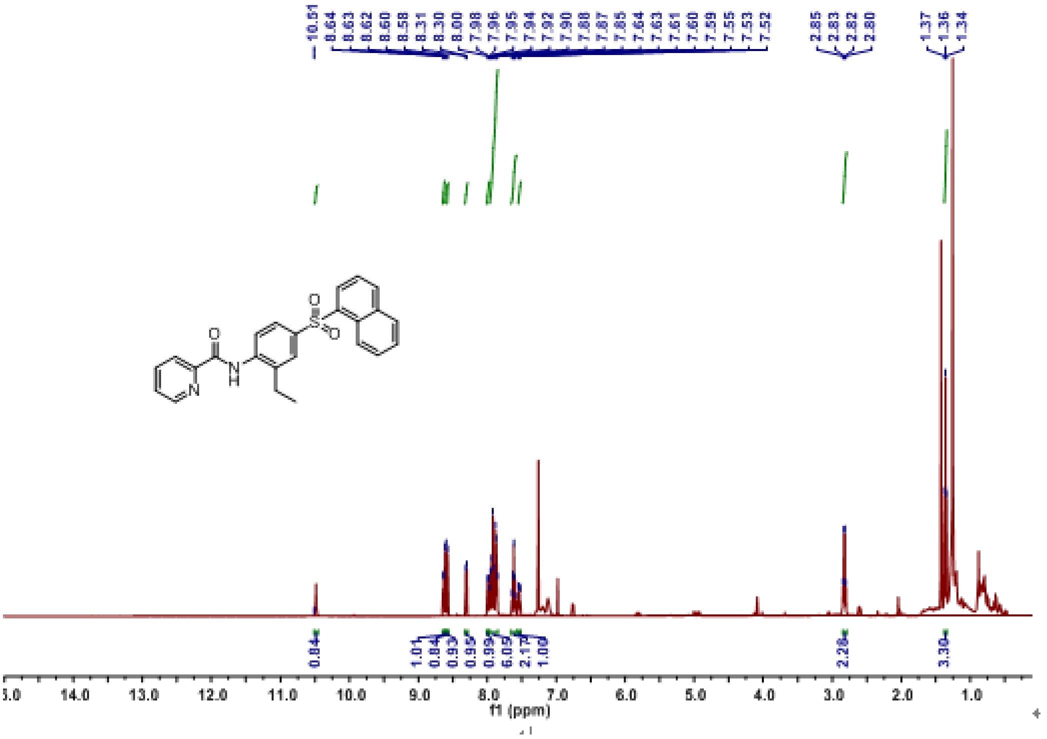

[1] **H NMR** Spectrum of compound **3x**

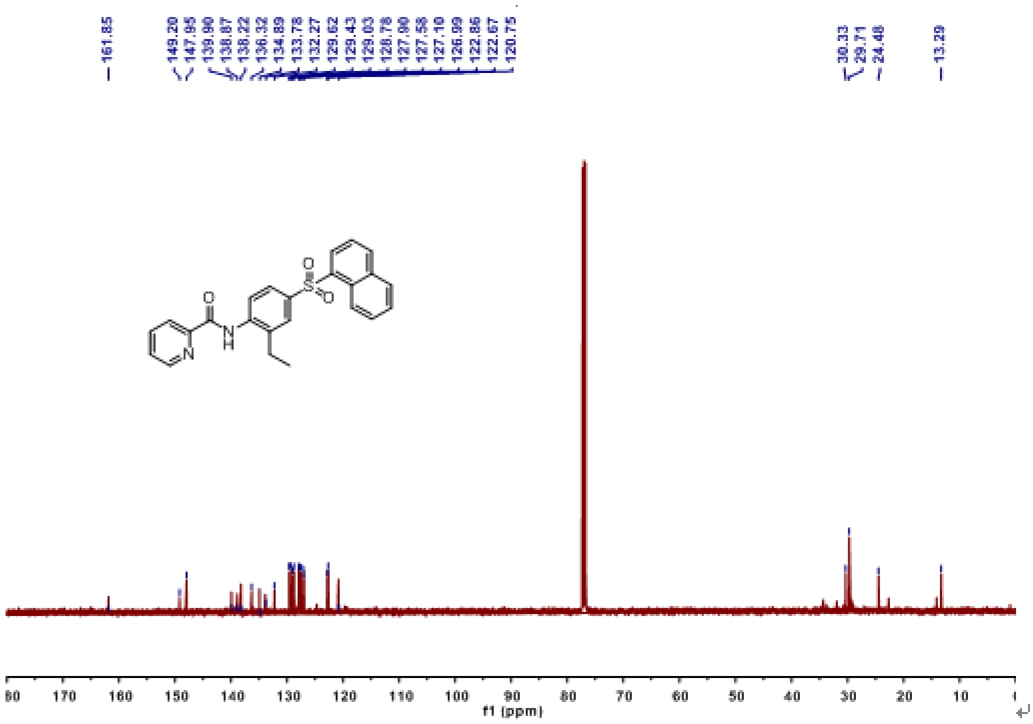

^{13}C NMR Spectrum of compound **3x**

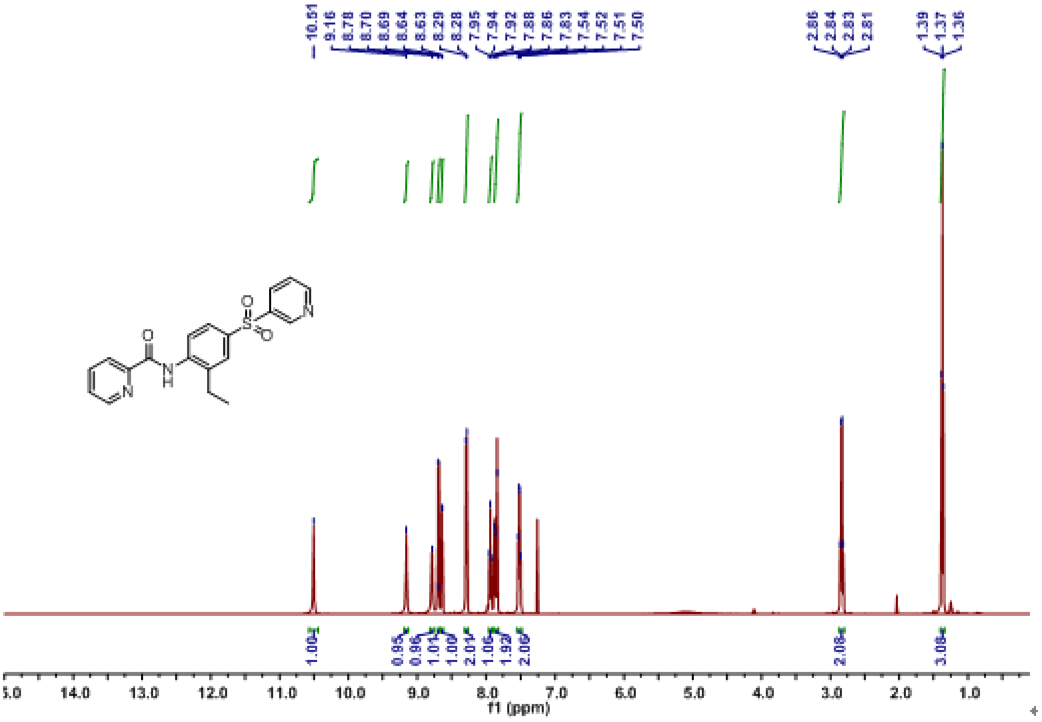

^{1}H NMR Spectrum of compound **3y**

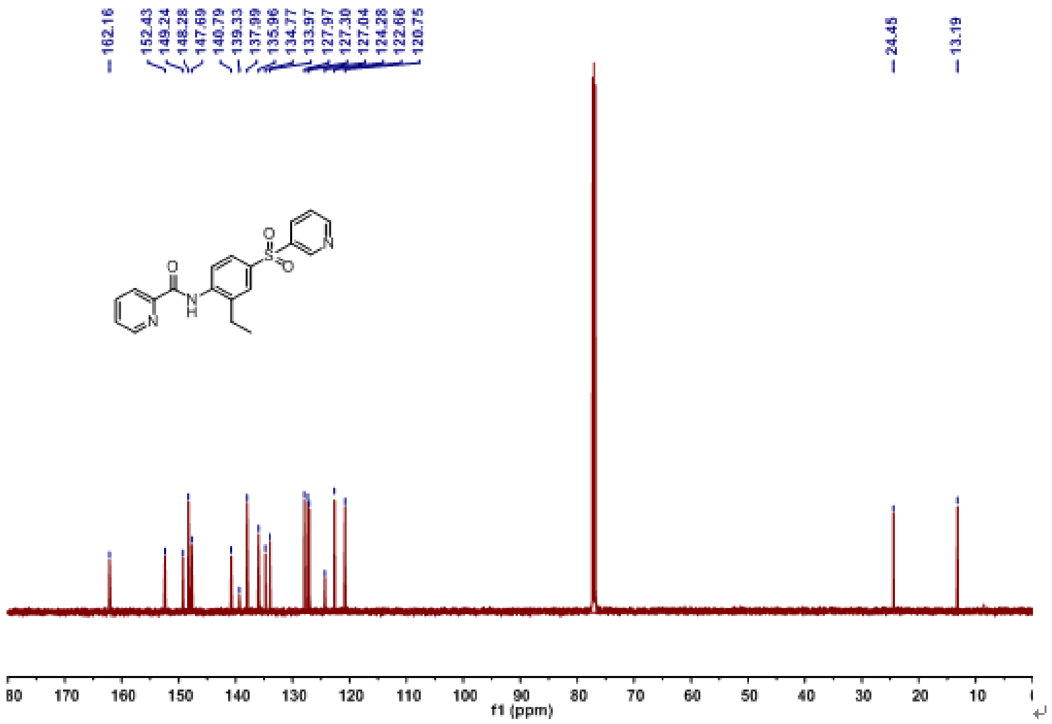

^{13}C NMR Spectrum of compound **3y**

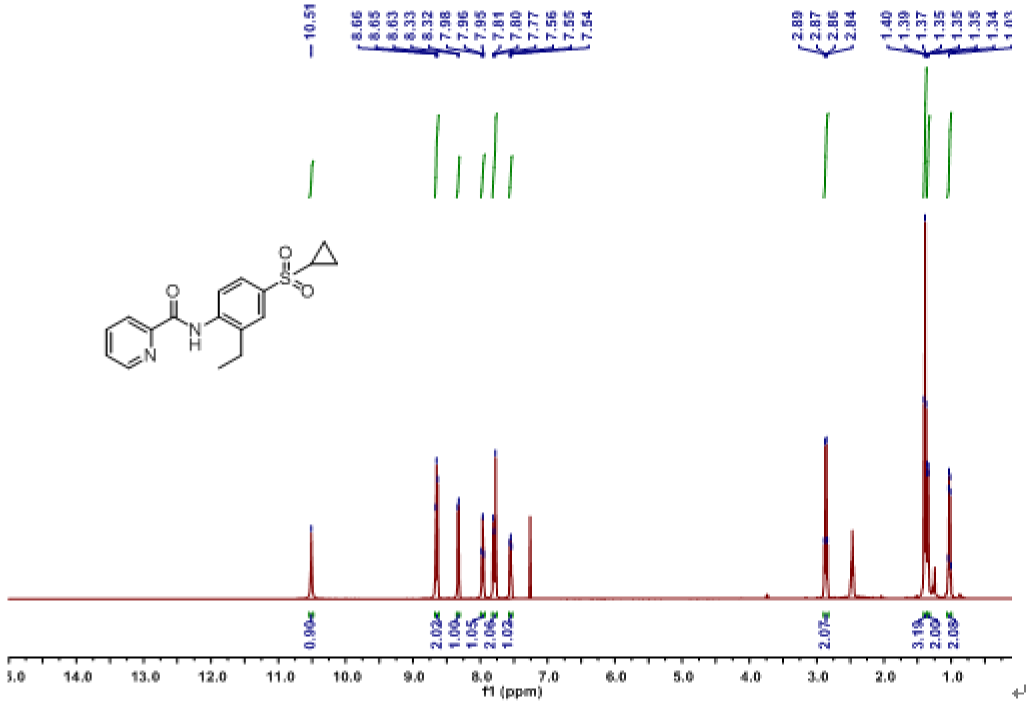

^{1}H NMR Spectrum of compound **3z**

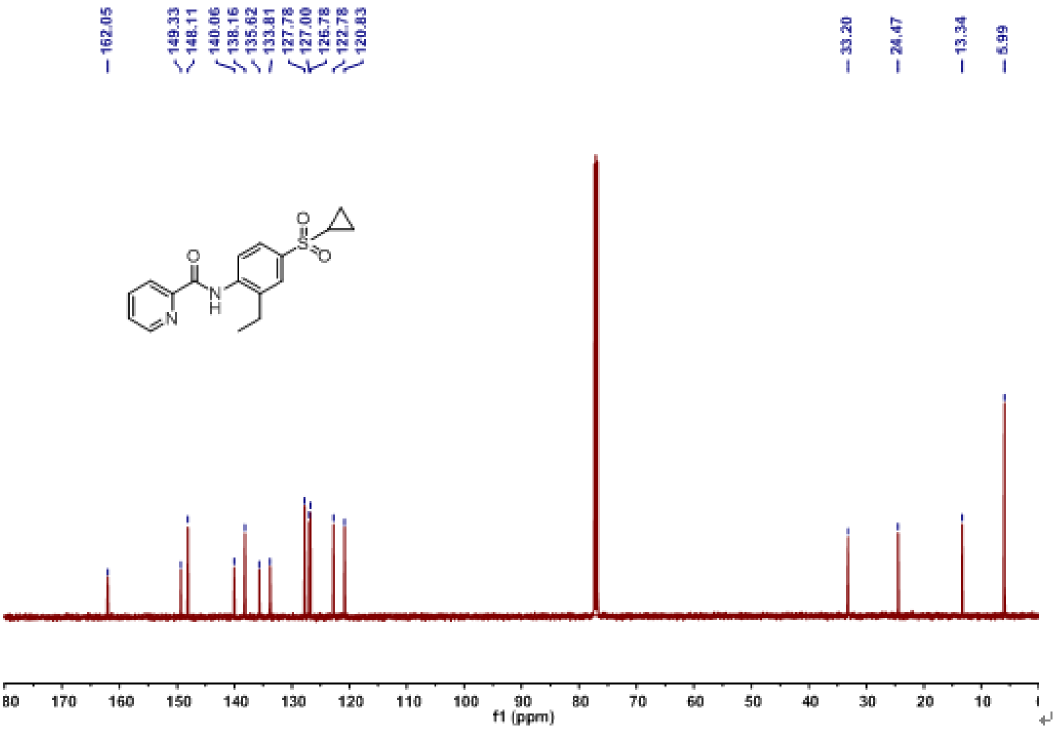

^{13}C NMR Spectrum of compound **3z**

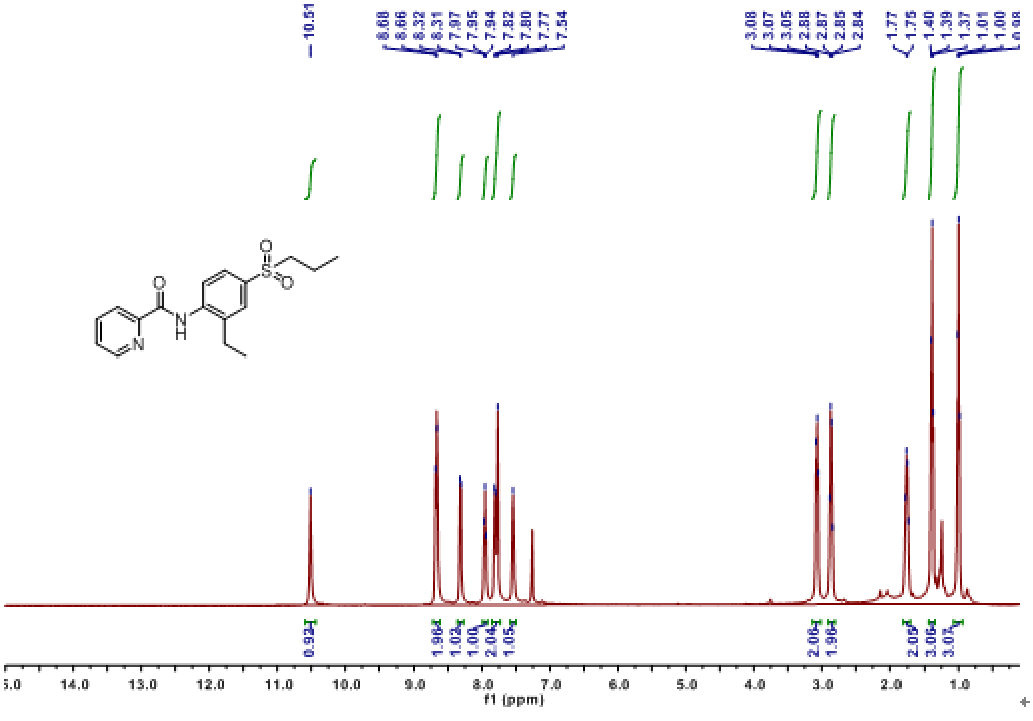

^{1}H NMR Spectrum of compound **3aa**

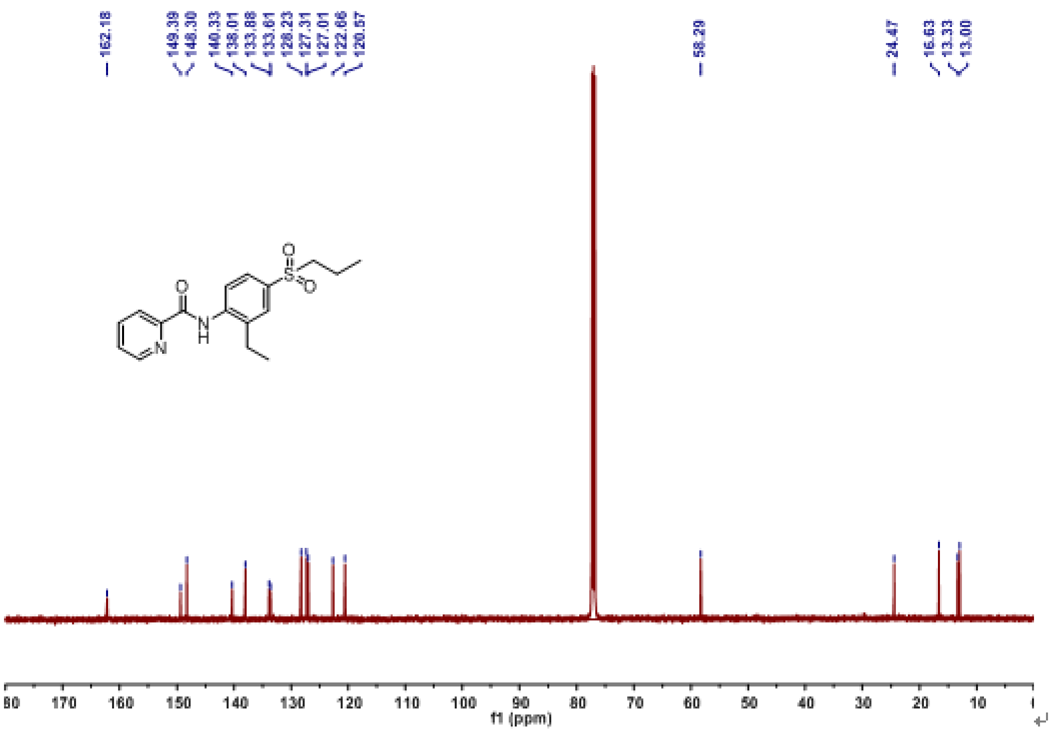

^{13}**C NMR** Spectrum of compound **3aa**

7. References

[1] Qiao, J.; Wang, T.; Zheng, K.; Zhou, E.; Shen, C.; Jia, A.; Zhang, Q. Magnetically reusable Fe_3O_4@NC@Pt catalyst for selective reduction of nitroarenes. *Catalysts* **2021**, *11*, 1219-1232, doi:10.3390/catal11101219.

[2] Liang, S.; Bolte, M.; Manolikakes, G. Copper-catalyzed remote para-C-H functionalization of anilines with sodium and lithium sulfinates. *Chem. -Eur. J.* **2017**, *23*, 96-100, doi:10.1002/chem.201605101.

[3] Bai, P.; Sun, S.; Li, Z.; Qiao, H; Su, X.; Yang, F.; Wu, Y.; Wu, Y. Ru/Cu photoredox or Cu/Ag catalyzed C4−H sulfonylation of 1-naphthylamides at room temperature. *J. Org. Chem.* **2017**, *82*, 12119-12127, doi:10.1021/acs.joc.7b01917.

[4] Zhou, X.; Yu, R.; Wang, J.; Liao, X; Xiong, Y. Copper-catalyzed remote sulfonylation of 1-naphthylamides with sodium-sulfinates. *Chin. J. Org. Chem.* **2021**, *41*, 4370-4377, doi:10.6023/cjoc202106030.

[5] Hangzhou Vocational & Technical College. Sulfonyl pyridine amide derivatives and its preparation method. *CN112142656 A.* **2020-12-29**.

[6] Sarkar, S.; Sahoo, T.; Sen, C.; Ghosh, S. C. Copper(II) mediated ortho C-H alkoxylation of aromatic amines using organic peroxides: efficient synthesis of hindered ethers. *Chem. Commun.* **2021**, *51*, 8949-8952, doi: 10.1039/d1cc01803e.
